# Asymmetric transfer of CO_2_ across a broken sea surface

**DOI:** 10.1038/s41598-018-25818-6

**Published:** 2018-05-29

**Authors:** Timothy G. Leighton, David G. H. Coles, Meric Srokosz, Paul R. White, David K. Woolf

**Affiliations:** 1 Institute of Sound and Vibration Research, University of Southampton, Highfield, Southampton, SO17 1BJ UK; 2 National Oceanography Centre, Southampton, SO14 3ZH UK; 3 Orkney Campus, Heriot-Watt University, Stromness, KW16 3AW UK

## Abstract

Most estimates of the climatically-important transfer of atmospheric gases into, and out of, the ocean assume that the ocean surface is unbroken by breaking waves. However the trapping of bubbles of atmospheric gases in the ocean by breaking waves introduces an asymmetry in this flux. This asymmetry occurs as a bias towards injecting gas into the ocean where it dissolves, and against the evasion/exsolution of previously-dissolved gas coming out of solution from the oceans and eventually reaching the atmosphere. Here we use at-sea measurements and modelling of the bubble clouds beneath the ocean surface to show that the numbers of large bubbles found metres below the sea surface in high winds are sufficient to drive a large and asymmetric flux of carbon dioxide. Our results imply a much larger asymmetry for carbon dioxide than previously proposed. This asymmetry contradicts an assumption inherent in most existing estimates of ocean-atmosphere gas transfer. The geochemical and climate implications include an enhanced invasion of carbon dioxide into the stormy temperate and polar seas.

## Introduction

**Figure 1:**
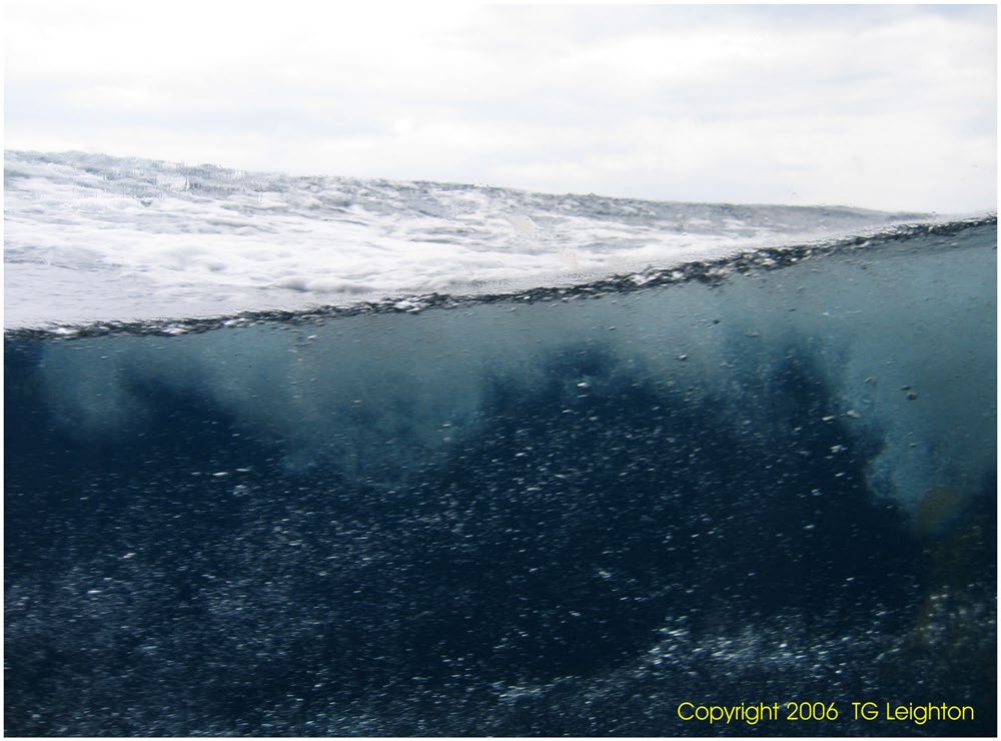
Photograph showing subsurface bubble clouds (taken by T.G.L.).

The role of the ocean in contributing to climate control and change has been recognized for many years. One aspect of that role is as a significant sink of anthropogenic carbon dioxide^1,2^, and a major source or sink of several other climatically-important gases. Calculations of air-sea flux of each gas depend on the estimation of exchange coefficients, whose values depend on wind-driven processes at the air-sea interface. Most estimates implicitly assume stirring across an intact sea surface^3–5^, but the broken surface (Fig. 1), characterized primarily by bubbles under breaking waves, should be considered^6–9^. Bubble-mediated transfer is inherently asymmetric^10,11^, with a bias towards injection into the ocean. Here we show that the numbers of large bubbles found metres below the sea surface in high winds are sufficient to drive a large and “asymmetric” flux of carbon dioxide in contradiction to previous studies. Extrapolation of this finding is shown to imply a substantial effect annually and globally.

## Gas flux

Estimates of the net flux of a gas across the sea surface^3–5^ generally assume an equilibrium based on Henry’s Law and the application of a Fickian diffusion equation, usually written in the form:

1 $$F=K({C}_{{\rm{a}}}-{C}_{{\rm{w}}}).$$

Here a net air-to-sea flux, *F*, of a gas species is proportional to the difference between the concentration of that species dissolved in the upper ocean (*C*_w_) and *C*_a_ (the liquid-phase concentration in equilibrium with the atmosphere, a feature which eliminates solubility from this and succeeding equations). For poorly soluble gases, the diffusion across the marine microlayer (the sub-millimetre layer of liquid immediately adjacent to the absolute sea surface) determines the exchange rate^5,12,13^. In a steady state most of the concentration difference is across this layer, implying that the molecular diffusion coefficient of the dissolved gas and the related non-dimensional Schmidt number, *Sc*, are key factors. Since there are few measurements of air-sea gas transfer velocities, *K*, most models of air-sea gas transfer^14^ assume a simple wind-speed dependence, scaling with Schmidt number (usually *K* ~ *Sc*^*−0.5*^) and a strict proportionality to air-sea concentration difference as implied by Equation (1).

If the surface is broken (e.g. by the generation of bubbles in breaking waves) then there will be a parallel pathway across these additional surfaces as gases transfer across the bubble wall. Moreover, the concentration difference driving this flux is different, since it depends on partial pressures in the bubbles, which will generally be raised due to the hydrostatic pressure on the bubbles and the effect of surface tension. In this case, Equation (1) is invalid for a bubble-mediated flux, but it is proposed^10^ that the following modified flux equation is suitable for the bubble-mediated flux, *F*_b_:

2 $${F}_{{\rm{b}}}={K}_{b}[({1}+\delta ){C}_{a}-{C}_{{\rm{w}}}].$$

Here, *K*_*b*_ is the contribution of bubbles to the air-sea transfer velocity of the gas, while *δ* describes an asymmetry in that transfer (see Supplementary Section S1 for a thorough explanation of the formulations used in this study). This bubble-mediated flux *F*_b_ occurs in addition to the flux directly across the sea surface of Equation (1). There are two key features of the model of Equation (2), which invalidate widely-held assumptions. Firstly, it is not credible to assume *K*_b_ will scale simply with Schmidt number^15^. This point implies that conventional extrapolations for one gas from another may be awry. The second key feature is an inherent asymmetry (embodied in *δ*) that favours injection into the ocean (i.e. favouring dissolution of gases into the ocean over the release of previously-dissolved gas from the ocean into the atmosphere)^8,10,11^. This point is potentially more far reaching, since the basic formula, Equation (1), used for most estimates of gas transfer is strictly incorrect and instead Equation (2) should be used for the bubble-mediated flux.

**Figure 2:**
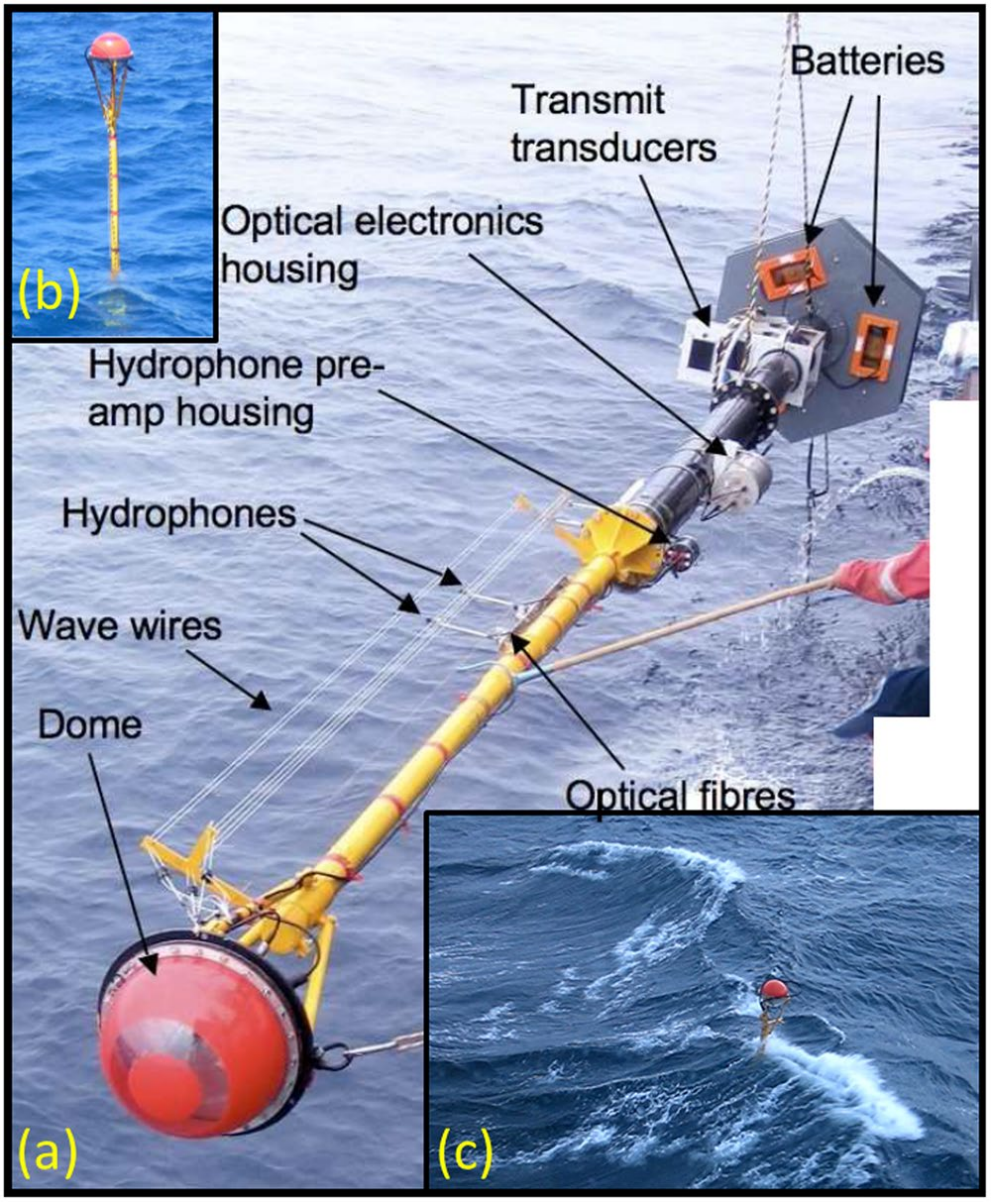
Photographs of the spar buoy being deployed and at sea. (**a**) The 11 metre long buoy being deployed from the ship (perspective makes the lower grey section appear shorter than the upper yellow section, although in reality it is nearly twice as long). (**b**) The buoy sitting in calm waters during the first cruise and (**c**) the buoy during the deployment described in this study. See also Fig. S2.3.

We validate Equation (2) and give estimates for the transfer coefficients *K*_*b*_ and *δ* by combining a model of subsurface bubble cloud evolution with measurements in the open ocean of the bubble size distribution (BSD, the histogram of bubble concentrations, as a function of radius) from a free-floating instrumented buoy (Fig. 2).

## Results

**Figure 3:**
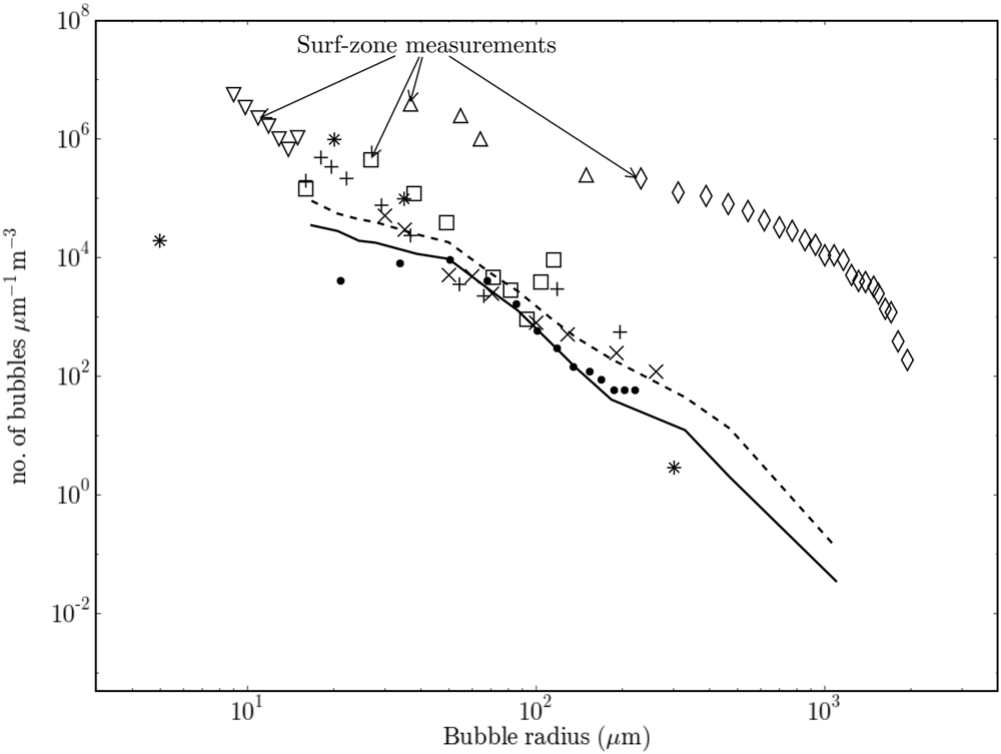
Bubble size distributions (BSDs) from this and historical studies. The BSDs measured in this study are shown by the broken and solid lines, measured at depths of 1.15 and 2 metres respectively. The graph compares these data with historical measurements. The historical data include the open ocean data of Breitz and Medwin^32^ (crosses), Phelps and Leighton^33^ (plus signs), Farmer and Vagle^34^ (stars) and Johnson and Cooke^35^ (dots), and the surf zone data of Deane and Stokes^16^ (diamonds), Phelps *et al*.^17^ (triangles), Meers *et al*.^36^ (downward pointing triangles) and Leighton *et al*.^22^ (squares).

The bubble size distributions measured in this study are presented together with some historical data sets in Fig. 3 (the technical and environmental parameters for each study are summarised in the Supplementary material). Much higher concentrations of bubbles have been measured in the surf zone^16,17^, but all the other studies report broadly comparable distributions. The novel observation is that substantial numbers of large (i.e. substantially larger than 100 µm radius) bubbles penetrate to 1 and 2 metres depth. The large values of δ reported below follow substantially from this observation.

**Figure 4:**
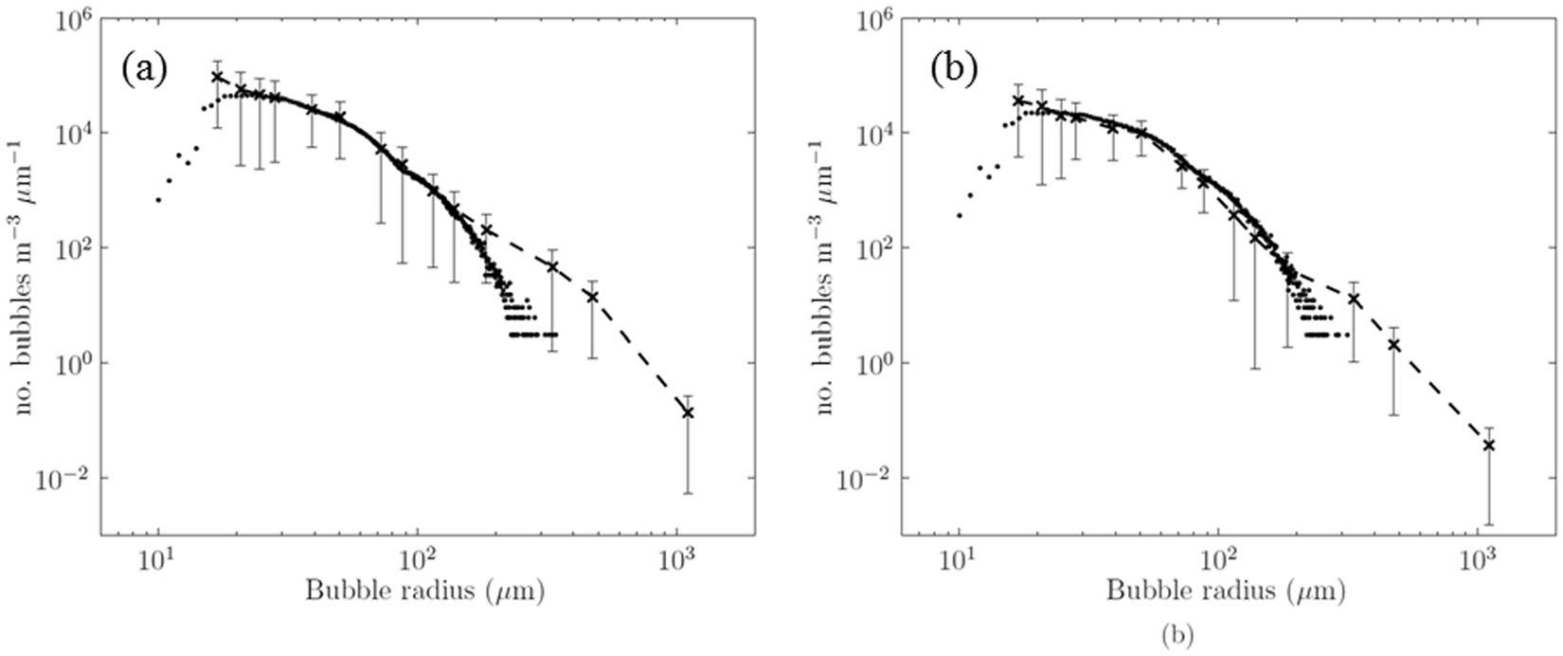
Bubble size distributions from the measured data (dashed line with crosses) and the model (circles). Panel (a) shows the distributions at 1.15 m depth and (**b**) shows them at 2 m depth. The uncertainty bars show one standard deviation from the mean within these data.

To quantify *K*_b_ and *δ* for individual atmospheric gases, the evolution of bubbles clouds under breaking waves and the resulting gas flux, was modelled. This modelling extends earlier work^10^ to apply more recent observations^18^ of the initial bubble size distribution (BSD) and the injection process. After the injection, the BSD changes over time as bubbles dissolve, expand or contract, as buoyancy and oceanic turbulence and circulation moves them to greater or lesser depths. Calculations of gas transfer across the surface of the bubbles are made for gases of interest. Outputs include instantaneous and time-averaged “modelled” BSDs. The time-averaged modelled and observed BSDs at the depths of the measurements in the Atlantic are merged to calculate the bubble-mediated gas transfer. Tuning of the model to the measurements is summarised under Methods and further detail is provided in Supplementary Section S3. The measured and modelled BSDs are shown in Fig. 4. Note especially that the measurement of bubbles is dependent on subtraction of a “baseline”, the attenuation of acoustic signals in the absence of bubbles. Uncertainty in that baseline carries through first to the measured BSDs and on to the modelled BSDs and gas fluxes. Generally, the fit of the model to the data after calibration is satisfactory, but is relatively poor for radii greater than 200 *μ*m. That remaining discrepancy is significant to the accuracy of our results and is considered in the Discussion.

**Figure 5:**
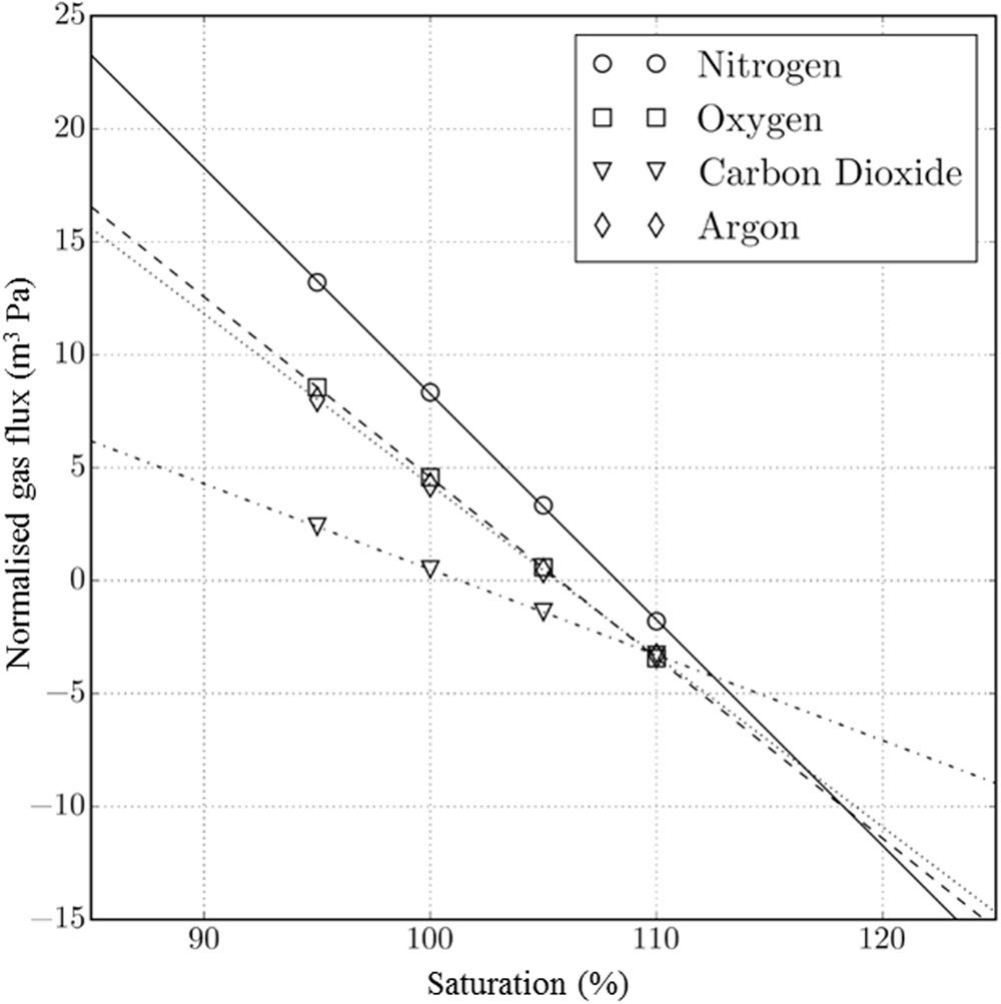
The modelled flux of bubble-mediated gas transfer plotted against saturation for the four gases considered. An injection of gas is predicted at saturation, while balanced at a supersaturation, *δ*. The fluxes have been normalised such that the values are proportional to the rate of change of saturation of each dissolved gas. Generally, both *δ* and the rate of change reduce with increasing solubility.

To quantify *K*_b_ and *δ* from this model, the mass flux parameters were evaluated in the following manner. With the measured ancillary data (detailed in Supplementary material) as input, the model was run with bubble injection by successive breaking waves until the bubble cloud reached steady state (i.e. when the variation of BSD with depth no longer changed significantly with each new injection). A range of fixed intervals between successive breaking wave events was tested, for example in the sensitivity study, but it was fixed at 8 s for production of the final results. Comparison of the steady state output with the time-averaged BSD found during the 10-hour at-sea measurements confirmed the validity of the model and allowed the values of a few remaining ancillary parameters (i.e. those which could not be measured directly in the trial; see Supplementary Section S3.3.3) to be estimated by calibration. Having determined all of the input parameters, the model is run again with bubbles injected only once, at the start of the simulation. This second type of simulation was run four times for each gas species in seawater, using four varying values of the concentrations varying from 95% to 110% of the saturation, $({C}_{{\rm{w}}}/S{p}_{{\rm{pw}}})\times 100 \%$, where *S* is the solubility and *p*_pw_ is the partial pressure of the gas in question in the water. As shown in Fig. 5, when *F*_b_ is plotted as a function of the saturation, the data follow the straight-line dependence implied by *F*_b_ = *K*_b_ [(*1* + *δ*)*C*_a_ − *C*_w_], thereby validating Equation (2). Furthermore, *K*_b_ and *δ* can be calculated from the gradient and intersect of the linear fit.

The calculated values of *K*_b_ and *δ* for the four gases modelled are summarised in Table 1. All values are substantial in the context of typical air-sea gas transfer rates. Coefficients are expected to vary among gases, since they will depend on the molecular diffusion coefficient of the dissolved gas and the solubility of the gas. In particular, both coefficients are expected to be lower for more soluble gases. This expectation is met by the results. Nitrogen, which is the least soluble gas, has the highest values of *K*_b_ (21.5 cm hr^−1^) and *δ* (8.27%), while CO_2_ has the lowest (*K*_b_ = 8.1 cm hr^−1^ and *δ* = 1.32%) Oxygen and argon, which are very similar in physical constants, are intermediate in both values. As noted already, uncertainty in an acoustic baseline introduces uncertainty and that carries through to estimates of gas flux. That implies an uncertainty in *K*_b_ that is represented by the minimum and maximum values in brackets in Table 1.

**Table 1:** Estimates of transfer velocity (*K*_b_) and the equilibrium supersaturation (*δ*) from this study and historical estimates for each of the four gases included in this study.

Gas	All data *K*_b_ (cm hr^−1^)	All data *δ* (%)	*H*_s_ = 1.9 m *K*_b_ (cm hr^−1^)	*H*_s_ = 3.1 m *K*_b_ (cm hr^−1^)	Woolf & Thorpe^10^ (1991) *Δ* (%)	Woolf^7^ (1993) *K*_b_ (cm hr^−1^)	Keeling^6^ (1993) *K*_b_ (cm hr^−1^)	Keeling^6^ (1993) *δ* (%)
Nitrogen	21.5 (13.8–31.0)	8.27	9.7	24.1	3.78	—	—	—
Oxygen	17.2 (11.0–24.8)	5.72	7.7	19.3	2.42	—	13.84	0.25
Carbon dioxide	8.1 (5.2–11.7)	1.32	3.7	9.1	0.0816	8.18	2.96	0.08–0.3
Argon	16.4 (10.5–23.6)	5.60	7.4	18.4	2.13	—	—	—

The first two columns are the final estimates from this study. Values in brackets are the minimum and maximum values of *K*_b_ based on uncertainty in the “baseline”. Two additional values of *K*_b_ are based on subsets of the data when significant wave height averaged 1.9 m and 3.1 m respectively. Historical estimates from literature^6,7,10^ are also shown. One of the values of supersaturation is for a different definition of supersaturation^10^, Δ, as explained in the text. All values are for a wind speed of 14 m s^−1^ (at 10 m above the sea surface).

**Figure 6:**
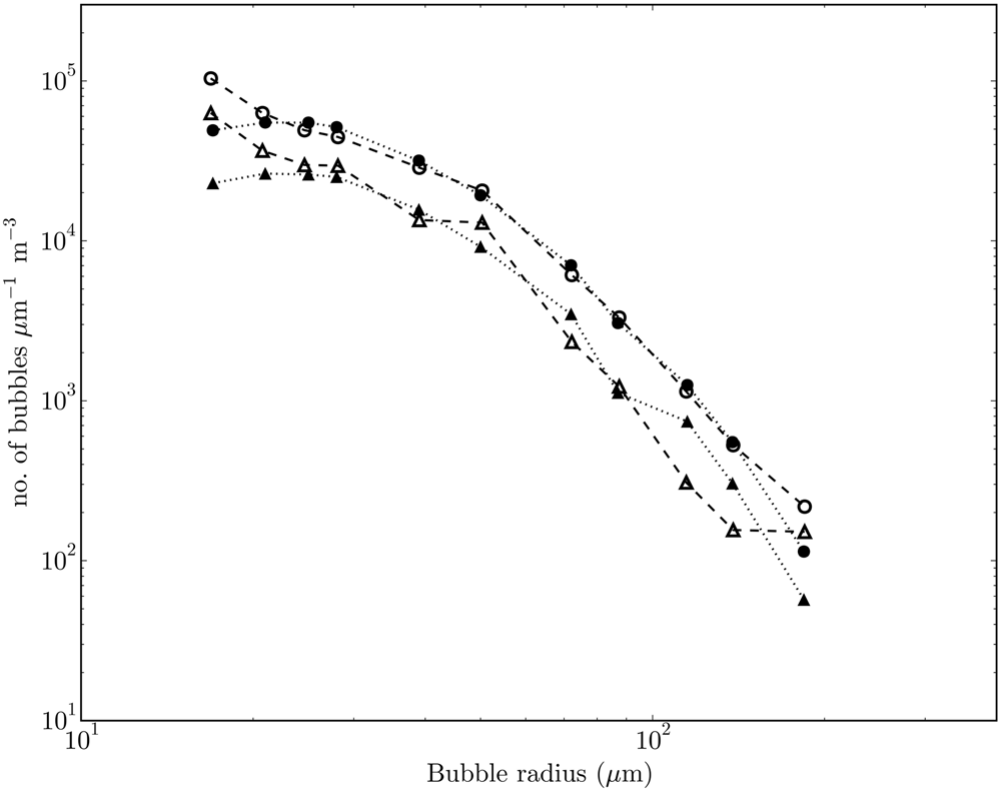
Measured and modelled bubble size distributions for mean significant wave heights of 3.1 m (circles) and 1.9 m (triangles). Measurements are shown by open circles (*H*_*s*_ = 3.1 m) and open triangles (*H*_*s*_ = 1.9 m). The model fits are shown by filled circles (*H*_*s*_ = 3.1 m) and filled triangles (*H*_*s*_ = 1.9 m).

Since the sea state grew during the complete measurement period, it was possible to obtain estimates of *K*_b_ for two different sea states. Two subsets of the full data set were analysed, the first one third and the remainder, corresponding to average values of significant wave height of 1.9 m and 3.1 m respectively. A marked difference in acoustic attenuation between these two sea states was measured, which is consistent with an increasing frequency of wave breaking (Fig. S3.9). Measured and modelled BSDs for these two subsets (at 1.15 m depth only) are shown in Fig. 6, in which the difference is readily apparent. The estimated concentration of bubbles varied substantially between the two subsets and from the full data set. In each case, the rate of bubble injection was tuned to provide the best fit between measured and modelled BSD. In the earlier period, injection rate was 45% of the standard set, while it was 112% for the later period. This rescaling translates to the values of *K*_b_ as shown in Table 1.

## Discussion

Estimates of the contribution of “bubble-mediated transfer” have previously depended on a postulated population of bubbles^6,10,11^. Those estimates are supported by estimates of gas transfer velocity for various gases^19^, but direct evidence of sufficient bubbles has been missing. This study represents a first opportunity to calculate gas transfer coefficients directly from an adequate observation of the bubbles responsible (albeit on a single day and location). The results support the view that in strong winds a substantial fraction of air-sea exchange is by bubble-mediated transfer and that transfer is strongly asymmetric.

The results support the use of Equation (2) to represent the asymmetric gas transfer. An additional specific term to describe total dissolution of some bubbles^8^ is not required, though some bubbles will have dissolved in the simulations. Table 1 includes a few key estimates of transfer coefficients from the literature, calculated for a wind speed of 14 m/s for easy comparison with our results. The values of *K*_b_ from this study are fairly high, but broadly consistent with previous estimates^6,7^ and support the hypothesis that there is a substantial enhancement of the total transfer velocity of gases by bubbles. The transfer velocities are sensitive to the choice of bubble-water transfer coefficients and also to the treatment of injection and mixing very close to the sea surface. Considering also the uncertainty of the baseline measurement (bracketed values in Table 1) and the difficulty of extrapolating from a single deployment in a building sea, the values of *K*_b_ generally support contemporary views of the significance of bubble-mediated transfer, but cannot narrow uncertainties.

The values of equilibrium supersaturation from our study are more remarkable and represent a significant new finding. We include two historical estimates^6,10^ in Table 1, but note that each of these estimates needs to be put in context. Woolf and Thorpe^10^ modelled the injection of a plume dominated by small bubbles, inconsistent with newer observations of the initial BSD. Significant asymmetry was found, but this is expressed as an asymmetry of the total transfer, Δ (which is less than δ, in the ratio of *K*_b_ to the total transfer velocity, see Supplementary Section S1). Keeling^6^ considered large bubbles, but only rising from a shallow depth. Our study is the first to assess the significance of large bubbles (i.e. bubbles substantially larger than about 100 µm radius) advected to depths of one or two metres. The following two paragraphs outline the evidence to support the hypothesis that the asymmetry reported here is a robust result and should supplant previous estimates.

**Figure 7:**
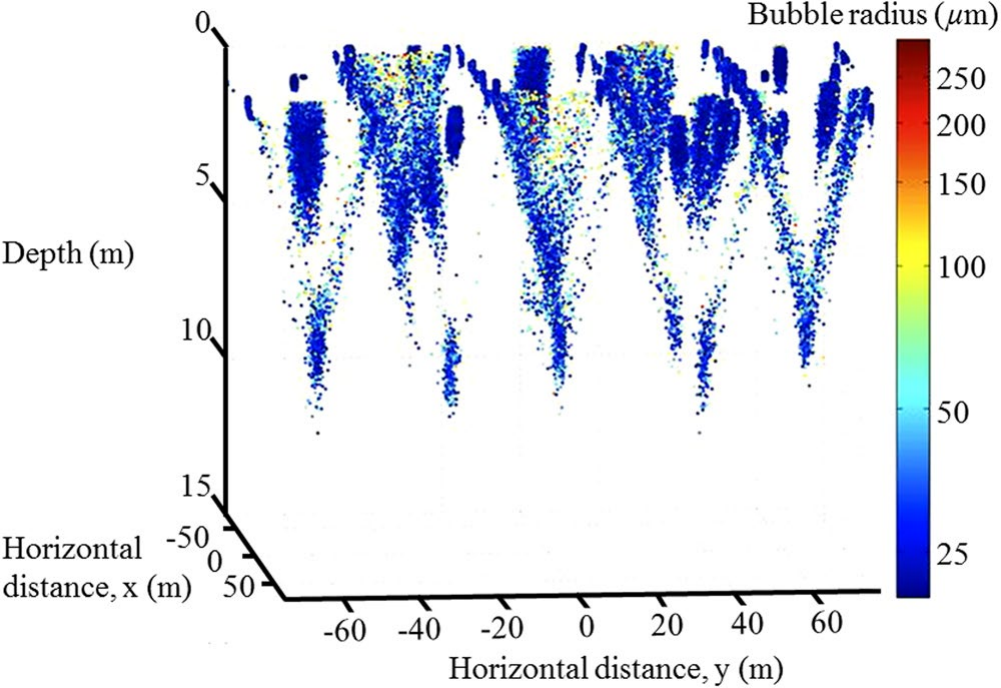
The bubble clouds at the end of the model run. The helical flow of the Langmuir cells can be seen. The population shown here is from a run with 100000 bubbles in each input population, and only 1 in every 100 bubbles is plotted (the larger bubbles being shown red, the smaller ones blue). Details of the input parameters can be found in Supplementary Section 3.

Substantial asymmetry of gas exchange has been demonstrated previously^8,9^ for Noble gases and oxygen (and by implication for other very poorly soluble gases) by measurement of their oceanic supersaturation. These values can also be validated by measurements of the small bubbles in deep clouds^10^. The fact that supersaturations exist indicates the importance of bubbles. Our study predicts supersaturations rising to very high values (e.g., 5.72% for O_2_, 5.60% for Ar), but only when bubble-mediated exchange is dominant. These values are consistent with net supersaturations of ~1% for more normal conditions where bubble-mediated exchange is only a fraction of the total air-sea exchange. The supersaturations of slightly more soluble gases (including CO_2_) are expected to be lower, but extrapolation from less soluble gases^8–11^ is inexact without accurate information on BSDs; existing estimates of *δ* for carbon dioxide assume a distribution of bubbles near the sea surface in windy conditions. It has been demonstrated that many large bubbles are injected to several metres depth in hurricane conditions^20^ and that bubbles can be driven to several metres depth through Langmuir circulation^21^ (Fig. 7), but prior to our new measurements the significance of a relatively deep penetration of large bubbles has not been appreciated. The concentration of bubbles up to a radius of 200 *μ*m could be simulated, but we could not find an acceptable fit for even larger bubbles. The modelled concentrations are lower than the acoustically-measured concentrations for the largest bubbles. Had this mismatch occurred for a population where all bubbles were much smaller than a wavelength in radius, then it would indicate a mismatch between the model and the real BSD. However it is not possible to have the same level of confidence when the sound field interacts with large bubbles, because then it is possible (but not provable given the optical system was damaged) that the model continues to match the true BSD, and the actual mismatch is between the acoustically-inferred BSD and the real BSD. This is because the accuracy of the estimation of BSD reduces when the sound field interrogates large bubbles. A key assumption of the acoustic model which estimates the BSD from the measured acoustic attenuation (the ‘inversion’), is that the bubbles are oscillating in steady state, and the maximum achievable pulse length may be insufficient to achieve that (see Supplementary Sections S2.1.1 and S3.4.1). Another key assumption in the inversion is that the product of the largest bubble radius and highest acoustic wavenumber are much less than unity^22,23^, (i.e. that all bubbles are much smaller than the smallest wavelength used; see Supplementary Section S2.1.2). For 1 mm radius bubbles, this value (at 197 kHz) equals 0.8, and the assumption becomes compromised, making the estimation less accurate for large bubbles. This is generally true for all active acoustic measurements of bubble size, and so to offset this limitation, an optical system^24,25^, of measuring bubble size was implemented on the buoy to make a complementary estimate of BSD for larger bubbles (optical systems show the opposite trend to that displayed by the acoustics, tending to have higher accuracy for larger bubbles). The plan was to extend the measurement of the BSD to even larger bubbles using this overlap, and to compare the optical and acoustic estimates in the overlapping region at around 1 mm radius to obtain a better estimate than either system gives alone. This planning was negated when deployment of the buoy damaged the optical system. Note that the modelled bubble concentrations imply the gas transfer coefficients reported in this study and if the modelled concentrations are too low, the transfer coefficients are also too low.

Though there are remaining discrepancies between the measured and modelled concentrations of large bubbles, we have shown that there are sufficient populations of large bubbles to support substantial values of *K*_b_. Moreover, we have found that many of these large bubbles penetrate at least 2 metres below the sea surface in windy conditions and this underpins predictions of large *δ* for carbon dioxide. Hydrostatic pressure (in addition to a lesser contribution from surface tension and surface curvature), increases the gas pressure in the bubble and is the origin of the asymmetry. One metre below the sea surface, the hydrostatic pressure equals 10% of atmospheric pressure, implying an associated 10% asymmetry in gas transfer by this effect alone. It can be understood from this simple model that the observation of sufficient bubbles at depths of one and two metres underpins the calculation that the asymmetry will be larger than previously supposed. It should be noted however that the composition of bubbles will change as the bubbles evolve especially in the smaller bubbles, which underlies the greater significance of large bubbles and necessitates the more detailed modelling employed in this study. If the model underestimates the number of very large bubbles at depth (as implied by the acoustic data), then the true asymmetry will be even larger. The findings from, and equipment generated by, this relatively inexpensive 2007 experiment were sufficient to justify a well-supported multi-centre follow-on study^26^, currently underway, part of which uses the equipment, models and reports^27^ produced by this study. In addition, the acoustical methods are also being incorporated into a large multi-centre programme on carbon capture and storage^28^.

This study provides data only for a single 10-hour-period in the open sea at a water temperature of 17 °C, when the wind speed was fairly steady at 14 ms^−1^ and there was a growing wind sea. The data and associated model provide strong evidence of substantial and highly asymmetric bubble-mediated gas transfer on this occasion. The data also implies that the sea state is significant, since bubble concentrations increased as the waves grew. It is clearly a challenge to extrapolate the results of this study to all wind speeds, water temperatures and sea states. However, it is useful to consider the global implications, especially with respect to the asymmetry of the air-sea transfer of CO_2_, since this study radically alters understanding in this respect.

The global disequilibrium for CO_2_ is small, currently estimated as a net flux into the oceans of 1.6 PgC^3^ on an annual exchange of 80 PgC^1,2^. Since we have found an asymmetry of >1% on a substantial part of the total flux, the climatology of carbon dioxide should be revised. Bubble-mediated exchange of carbon dioxide may account for a quarter of total air-sea exchange^29^. An asymmetry of 1.32% in the bubble-mediated transfer imply an asymmetry of ~0.3% in the total or an additional annual downward global flux of 0.2–0.3 PgC. (This is an extrapolation from one 10 hr, high wind, single-location measurement, because experimental and funding challenges precluded obtaining more data (see Supplementary Section S2). The previous wisdom that the asymmetry was negligible is also based on extravagant extrapolation, but of less directly relevant information, as described earlier in this section). The additional flux is proportional to the partial pressure in the atmosphere^11^ and will increase with rising atmospheric carbon dioxide. Regional and seasonal effects will be greatest for relatively stormy localities such as the wintertime temperate and polar seas.

## Methods

Details of the Methods are to be found in Supplementary materials, where after exposition of the revised formulation for the transfer of gas across a broken ocean surface (Supplementary Section S1), details are given of the sea trials (Supplementary Section S2) and the bubble cloud and gas-flux modelling (Supplementary Section S3). In this section, a summary of the methods used in the sea trials are followed by some notes on the gas-flux modelling.

The BSD was measured at two depths using the acoustic attenuation between three hydrophones (having depths below the mean sea surface of 0.8, 1.22 and 2.54 m) vertically aligned on a 11 m long spar buoy^24,25^ deployed in the North Atlantic (43.1°N, -17.7°W) on 28 June 2007. The BSD was determined by inverting the acoustic attenuation of a sequence of 14 tones (at frequencies ranging from 3 to 197 kHz) projected upwards from the base of the buoy once every second. The BSDs reported in this paper cover bubble radii from 16 to 1141 *μ*m, the widest size range ever measured at sea. The measurements of BSD are dependent on subtraction of a “baseline”, the acoustic attenuation without bubbles. This baseline is an at-sea measurement, but introduces uncertainty that can be followed through to the calculated BSDs and on to the gas fluxes. A best guess, a minimum and a maximum baseline were identified and each was used in the analysis.

Additional data, including wind speeds, wave heights, video observation of wave breaking frequency and a segment of data from an inverted echo sounder showing the structure of bubble clouds (see Supplementary Sections S2 and S3), provide context and support for the measurements of BSD. During the selected period, water temperature averaged 17 °C and significant wave height increased steadily (a building wind sea) from <1.8 m to >3.5 m. An inferred contribution of bubbles to the measured acoustic attenuation is supported by measurements from an inverted echosounder (IES). For 10 minutes during the 10 hour measurement period, the IES at the base of the spar buoy monitored the position of the sea surface relative to the base of the buoy, and measured the profile of the bubble cloud through the backscattered signal (Supplementary material, Fig. S2.5). The IES revealed that the bubble clouds often penetrated deeper than the hydrophones. The IES data can validate the bubble population, its variation with depth and suggest the shape of bubble clouds advected across the sonar beam, but the full potential of the IES was not realised in this study, owing to the relative timing of measurements by hydrophones and IES. Nevertheless, as shown in Supplementary Section 2, the IES does provide valuable context for the attenuation measurements. In future studies, simultaneous IES and optical measurements (of large bubbles) would complement acoustic attenuation measurements of BSD, providing a fuller description of sub-surface evolution of the bubble clouds: although we achieved the measurement of the largest range of bubble sizes achieved at sea, the absence of optical data through instrument damage meant that key questions of the accuracy of the acoustically-inferred BSD for large bubbles, and how it affects our conclusions, could not be answered. The resulting uncertainty means that this paper sets a lower limit on the estimation of the asymmetry.

This experiment was part of a larger cruise plan^25^ aimed at parameterizing processes that influence aerosol production and the atmospheric content of radiatively important gases, including CO_2_. The wind speed plateaued in the afternoon and evening of 29 June 2007, fluctuating over 10 hours around an average of 14 m/s (i.e. windy, but not exceptional conditions). Data exclusively from this period were analysed for this study (the full set of sea trials are described in Supplementary Section S2; later studies by other investigators using our instrumentation, model and codes have not yet reported any data against which we can compare the results of this study).

The experimental data are then incorporated into the model of the evolution of bubble clouds beneath breaking ocean waves. The model is calibrated to the measurements by modifying the rate of bubble injection and “tuning” parameters that affect the penetration of bubbles to the measurement depth. Note that while we tuned the model, this was restricted to maintain the integrity of the model. The initial size distribution was set, since this is fairly established^18^. Parameters that were considered “tuneable” (within predefined limits) are the maximum upwelling/downwelling velocity within the Langmuir circulation, the initial input jet velocity, the time for the jet velocity to reach zero after injection, the insertion depth for the bubbles and the turbulent diffusion coefficient, but in each case, we started at a standard value and altered the values cautiously. The bubble distribution is especially sensitive to velocities within the simulated Langmuir circulation. A fairly high (but sensible) maximum velocity of 0.185 m s^−1^ appeared optimal. More details are described in Supplementary Section S3 including a tabulation of the final parameter values.

Variations in the measured BSD are associated both with the chosen baseline and with the two subsets of data in a rising sea state. Each alternative data set was analysed in the same way. The model was set to the final parameters found for the entire data set, but the number of bubbles was varied to fit the specific BSD.

The model of the evolution of bubble clouds includes transfer of gases across the surface of the bubbles. Therefore, once tuned the model also provides estimates of gas flux, which with appropriate scaling provide the estimate of gas transfer coefficients. Most of this part of the model is directly taken from Woolf and Thorpe^10^, but using more capable computing technology. Again, the details are provided in Supplementary Section S3.

The model of gas transfer across the surface of the bubbles assumes that the process depends only on molecular diffusion and the flow around the bubbles^10^. Among the processes that are excluded is chemical reactivity. It is known that CO_2_ is reactive with seawater, but the initial reaction is often assumed to be slow enough compared to the time scales of diffusive transfer across the sea surface to ignore^30^, except in very light winds. Since the transfer across the surface of a bubble is relatively quick (apparent by comparing “individual bubble transfer velocities”^10^ to the typical transfer velocity at the sea surface), it appears to be a safe assumption to ignore chemical reactivity in bubble-mediated transfer of CO_2_. However, it is worth noting that the rates of reaction and CO_2_ transfer at the sea surface could be raised by enzymes^31^ and this is possible also on the surface of bubbles.

The datasets generated during and analysed during the current study are are openly available from the University of Southampton repository at http://dx.doi.org/10.5258/SOTON/D0492.

## Electronic supplementary material

Supplementary material

Waves on deck of Discovery

Rolling ship

Wave_passes_buoy

Langmuir animation
