# Supplementary material for: Asymmetric transfer of CO2 across a broken sea surface
Source: Sci Rep. 2018 May 29;8:8301. doi: 10.1038/s41598-018-25818-6 (PMC5974314; doi:10.1038/s41598-018-25818-6)
Supplement: Supplementary file 1 — Supplementary material [file 41598_2018_25818_MOESM1_ESM.pdf]

Supplementary material (3 sections) to the manuscript
‘Asymmetric transfer of CO_2 across a broken sea surface’

Timothy G. Leighton[1*], David G. H. Coles[1], Meric Srokosz[2], Paul R. White[1] & David K. Woolf[3],

[1] *Institute of Sound and Vibration Research, University of Southampton, Highfield, Southampton SO17 1BJ, UK*

[2] *National Oceanography Centre, Southampton, SO14 3ZH, UK*

[3] *Orkney Campus, Heriot-Watt University, Stromness KW16 3AW, UK*

[*] Corresponding author: T.G.Leighton@soton.ac.uk; Phone: +44 23 80 592331

The figures for each section occur after the text for each section.

Contents:

Section title	Page range
Supplementary Section 1: Air-sea flux equation and transfer coefficients for the air-water transfer of gases	1.1 - 1.5
Supplementary Section S2 – Sea Trials	2.1 - 2.21
Supplementary Section 3: Bubble-Cloud and Gas-Flux Modelling	3.1 - 3.33

Supplementary Section 1: Air-sea flux equation and transfer coefficients for the air-water transfer of gases

S1.1 Standard Formulae

In the main paper, we have written the traditional formula for net air-sea gas fluxes in the simplified form

$F = K\,(C_a - C_w)$. (S1.1)

In this version, the role of solubility is hidden and we are describing the flux as being driven by a concentration difference, with a "rate constant", K. A more complete description can be found in the literature[37] and is summarized here.

We will start with a fairly general case of the free transfer of a gas between gas and liquid media. In the absence of chemical reactions, net gas fluxes per unit interfacial area between the gas phase, "g", and the liquid phase, "l" are dictated by the solubility of the gas, often defined through the dimensionless Henry's Law constant, K_H, and by turbulent and molecular processes that disperse gases across boundary layers on either side of the gas-liquid interface. If we define the concentrations on each side of the interface at a convenient reference level (a height or depth; or surface of constant concentration), then the flux equation can be written in two forms.

$F = -K_g(C_g - K_H.\ C_l) = K_l(C_g / K_H - C_l)$. (S1.1)

Here the effectiveness of all the dispersive processes between the two points is described by either K_g, written to emphasise gas-phase concentration differences and dispersion processes, or K_l, written to emphasise liquid-phase concentration differences; where C_g and C_l are the gas-phase and liquid-phase concentrations at the defined levels. Often "bulk-phase concentrations" are referred to in the literature, which presupposes that somewhere in the liquid and gas the concentration gradients are minimal and thus that in each medium there is a large "bulk" with a single concentration of the gas, but the formulae are more general if we define reference levels. If we separate the dispersion processes into processes in the gas phase represented by k_g and in the gas phase represented by k_l (each defined with respect to the concentration difference in the respective medium between interface and reference point), then it can be shown that:

$K_g = [1/k_g + K_H / k_l]^{-1}$, (S1.2)

$K_l = [1/(K_H\, k_g) + 1/k_l]^{-1}$. (S1.3)

Since transfer between the two reference points requires dispersion serially within both media, the slower of these processes largely determines the transfer rate. In this paper, we are primarily interested in the case $K_H\, k_g \gg k_l$, which applies to high K_H (low solubility), in which case

$K_l \cong k_l$, (S1.4)

$F \cong K_l (C_g/K_H - C_l)$. (S1.5)

Note that in this case, several parameters of the gas phase (including concentration differences within that phase, the precise magnitude of dispersion processes and the reference height above the sea surface) are insignificant; it is only necessary to define a concentration in the gas phase or equivalently the concentration at the interface. Thus we can translate between Equation S1.5 and Equation 1 in the main paper (reproduced here as Equation S1.1) by substituting "C_a" for "C_g/K_H", where C_a is the concentration in the liquid phase at the interface. (This concentration is assumed to be in equilibrium, by Henry's law, with the atmosphere). It is then only necessary to recognise that $K = K_l \cong k_l = k_w$, where k_w describes dispersion processes immediately beneath the sea surface; and furthermore that the approximation is adequate for poorly-soluble gases (including carbon dioxide) in the sea; and that $C_l = C_w$, the concentration in the oceanic mixed layer (in practice for air-sea exchange, we have to estimate a "bulk concentration" in the upper ocean that is used for calculations). Thus while we write about a general air-sea transfer velocity, K, we are really concerned with processes that contribute to k_w. For an unbroken sea surface, these processes are the combined action of molecular diffusion and turbulence that disperses gas between the interface and the relatively homogenous "mixed" water beneath.

S1.2 Revised Formulae for a Broken Sea Surface

We will limit discussion to processes that contribute[38] to "k_w". These processes are particularly relevant to poorly-soluble gases whose air-sea transfer is limited almost entirely by transfer on the liquid side of the interface (soluble gases are limited by transfer across the marine atmospheric boundary layers). A broken sea surface complicates matters, because in addition to dispersion by molecular and turbulent diffusion, gas may also be transferred *via* bubbles; that is, in the process of transfer the gas resides within bubbles for a period. This alternative mechanism is different in nature to the normal dispersive process as a result of the additional pressure on bubbles[39] and the finite capacity of the bubbles[40]. We wish to distinguish the flux directly across the sea surface, F_o, and the flux mediated by bubbles, F_b. The two parts of the flux can be written.

$F_o = K_o (C_a - C_w)$, (S1.6)

$F_b = K_b [(1 + \delta) C_a - C_w]$, (S1.7)

where the term, δ, defines the essential asymmetry of the bubble-mediated process.

The total flux, $F_T = F_o + F_b$, can be written in a number of ways[40], e.g.

$F_T = K_o (C_a - C_w) + K_b [(1 + \delta) C_a - C_w]$, (S1.8)

or

$F_T = K_T [(1 + \Delta) C_a - C_w]$, (S1.9)

where $K_T = K_o + K_b$ and $\Delta = \delta (K_b / K_T)$.

Moreover, it is possible to write the flux as the difference of an "invasion" flux into the sea and an "evasion" out, thus:

$F_T = K_i C_a - K_e C_w$, (S1.10)

where the asymmetry of the process is described by $K_i > K_e$ and Equations S1.9 and S1.10 are equivalent if $K_T = K_e$ and $\Delta = (K_i - K_e)/ K_e$.

We note these different forms for the same equation, in order to facilitate comparisons with the scientific literature.

Note first that the vast majority of studies implicitly assume that $\delta = 0$ and $K_T = K_e = K_i$. All the empirical parameterizations for poorly-soluble gases in common usage effectively apply to the "total transfer velocity", K_T, which is simply expressed as a single K, k_w or K_w (= K_l in Equation S3 but applied to the sea) usually with no acknowledgment of the separate fluxes. Most of the field determinations of transfer velocity are actually measuring K_e, but this is not universal. Only a very few studies have tried to separately estimate K_o and K_b and fewer still estimate δ or Δ. It is very important to note that while K_b may be ignored, it is the total transfer velocity, not just K_o that is inevitably measured and parameterised. (The difficulty with empirical parameterisations of k_w is that "bubble physics" is ignored in estimating the transfer velocity of one gas from measurements of another gas. That problem is significant[41], but here we will focus on the neglect of asymmetry). Asymmetry is routinely neglected and biases in the net flux of gases result. Broken sea surfaces also generate aerosols that can contribute to air/sea fluxes of mass, momentum and energy. This project was specifically designed to take RSS Discovery to encounter regions where the sea surface was broken by wave activity (Figure S1.1).

References for Supplementary Section 1

37 Liss, P. S. & Slater, P. G. Flux of gases across the air-sea interface. *Nature* **247**, 181–184 (1974).

38 Blomquist, B.W., Fairall, C.W., Huebert, B.J., Kieber, D.J. & Westby, G.R., DMS sea-air transfer velocity. Direct measurements by eddy covariance and parameterization based on the NOAA/COARE gas transfer model. *Geophys. Res. Lett.* **32**, 10.1029/2006GL025735. (2006).

39 Woolf, D. K. & Thorpe, S. A. Bubbles and the air-sea exchange of gases in near-saturation conditions. *J. Mar. Res.* **49**, 435-466 (1991).

40 Woolf, D. K. *et al.* Modelling of bubble-mediated gas transfer: Fundamental principles and a laboratory test. *J. Mar. Syst.* **66**, 71-91 (2007).

41 Jeffery, C.D, Robinson, I.S. & Woolf, D.K. Tuning a physically-based model of the air-sea gas transfer velocity. *Ocean Modelling* **31**, 28-35 (2010).

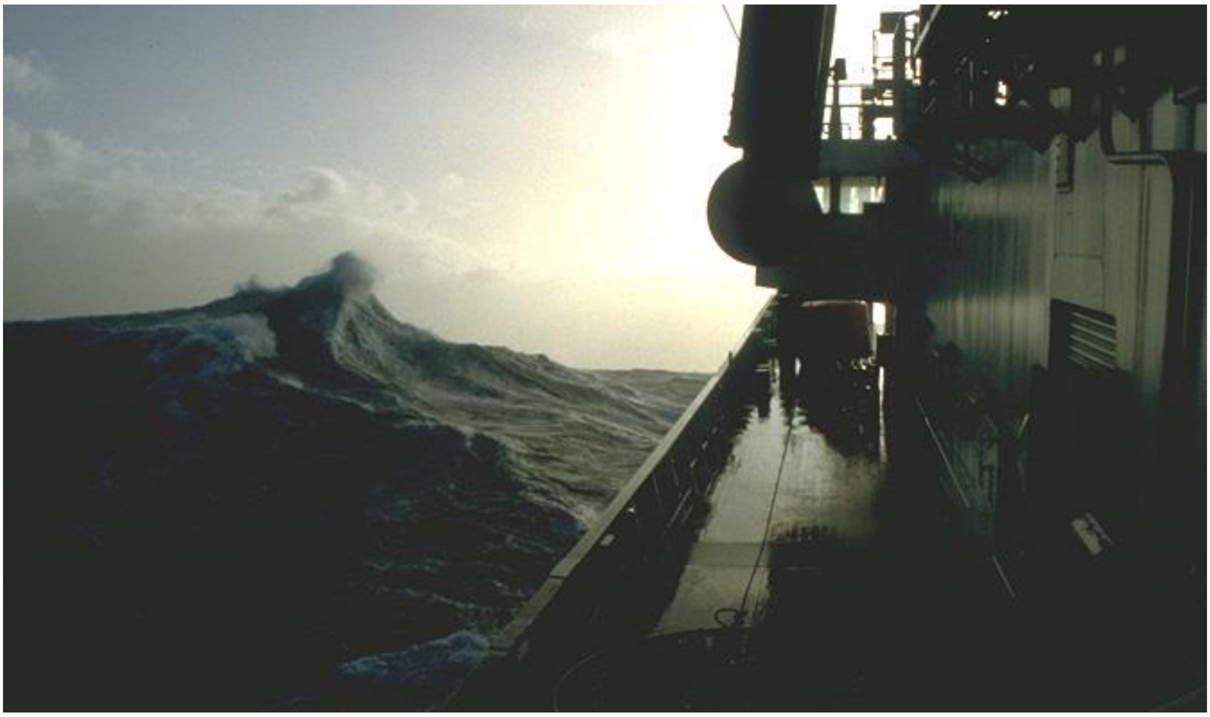

Figure S1.1 View of broken sea surface from *RSS Discovery* during the voyage.

Supplementary Section S2 – Sea Trials

As described in the main paper, our findings are based on unique measurements of the bubble size distribution (BSD) in the upper ocean, combined with data on the shape and depth of bubble clouds obtained by an upwardly-looking sonar. This section details the acoustic method for producing the necessary data, the spar buoy used to house or mount all instrumentation, the equipment necessary for measurement at sea and details of the successful deployment. Fuller details have been made available.[42]

This supplementary section focuses on the acoustical measurements of bubble size distribution. Because the bubble resonance frequency varies roughly inversely with bubble radius, acoustic techniques are good at measuring small bubbles, but become too expensive to use for the larger bubbles (since with today's technology each octave in the frequency of the sensor costs a similar amount, so that it costs roughly as much to measure bubbles resonant from 500-1000 Hz as it does to measure bubbles from 50-100 kHz)[43,44]. The low frequency acoustical limit that is consequently imposed is mitigated by the decreasing numbers of these bubbles found in the background oceanic bubble population. It is therefore sensible to combine acoustical measurements of the BSD with optical ones, since the latter more easily capture the large bubbles whilst failing to resolve the smaller ones (which is mitigated by the use of acoustics). The budget did not allow for high quality commercial optical systems, and therefore the spar buoy was equipped with inexpensive home-made prototype optical sensors[45]. Although these prototypes[46] measured bubbles from 50 microns radius to over 1 cm radius, the quality of the data was not sufficient for it to be combined with the higher-quality acoustical data that was used in the main paper, which nevertheless provided measurements on the largest range of BSD ever recorded at sea (bubble radii from 16 to 1141 μm). As with the optical measurements, funding was also not secured for resonator-type[47] acoustical systems that had been planned for this deployment, to provide complementary BSDs for comparison. Colleagues were however able to deploy on the spar buoy in-air downward looking video and stills digital cameras to obtain images of the sea surface, and capacitance wave wires and accelerometers to measure surface elevation and wave breaking.[46]

S2.1 Acoustic Method

The basic principle of the method is that the BSD may be inferred from the attenuation of an acoustic signal.[48] The signal was projected upwards from the base of the spar buoy, approximately once every second. The BSD between two hydrophones can be inferred from the attenuation of the signal between those hydrophones (here spaced 0.42 and 1.32 m apart, the spacing dictated by suitable mounting positions on the spar buoy itself). Ideally many hydrophones would be spaced along the length of the spar buoy, but here the budget allowed only three

hydrophones positioned at different depths along the spar buoy[49]. The BSD is inferred at two depths from each pair of neighbouring hydrophones. The key to the method is the construction of a suitable signal and the inversion of that signal to estimate the concentrations of bubbles within a particular bubble radius range, and constructing an estimated BSD by incrementally changing that size range. This is done by incrementally changing the centre frequency of each pulse in a train of 14 pulses (Table S2.1), and measuring the attenuation of each pulse in the train. The design of this train of pulses is discussed in the next section.

S2.1.1 Pulse design

The duration, shape and duty-cycle of the acoustic pulses were important factors to consider in the design of the experiment. One of the assumptions made in the formulation of the complex wavenumber[48] is that the bubbles are pulsating at the drive frequency of the pulse which excites them. The attenuation measured in the field will not be generated by such conditions if (i) the bubbles pulsate nonlinearly (a complementary model[48] was used to show this was not the case for this experiment) or (ii) the transient period before steady state pulsations are attained dominates the attenuation caused by the bubble over the duration of the pulse. Modeling the attenuation in this ring-up period[48,50] indicated that pulses should be at least 20 cycles in length[51,52]. One of the hydrophones was mounted within a metre of the surface (mean depth of 0.8 m) and surface reflections will interfere with the direct acoustic signal after a delay determined by the path length of the reflected signal. Therefore the pulse duration must be shorter than that delay and was a compromise of 1 ms, which meant that bubbles with a resonant frequency below 20 kHz would be excited for less than 20 cycles.

It is also necessary to allow the bubbles to ring-down after excitation.[48] This allows the experimental conditions at the start of each pulse to match the starting conditions of the theory (i.e. the bubble wall is at rest at its equilibrium position). Therefore a pause of 20 ms was left between each pulse in the pulse train.

The recorded waveform was likely to have noise contaminating the signal and therefore hinder the identification and analysis of each pulse. In order to help identification, a Gaussian window was applied to each pulse. The resulting pulse took the form shown in figure 2.1(a), with figure 2.1(b) showing the entire pulse train made up of 14 such pulses with the centre frequency incremented as shown in the figure.

S2.1.2 Frequencies for attenuation measurements

One of the goals of this experiment was to have bubble size distribution measurements over a large range of bubble radii. In order to achieve this, a broad range of frequencies needed to be used in the attenuation experiment. A target range of 2-200 kHz was set, as this covers the majority of the range measured by previous experimentalists, yet is broader than the measurements taken in any other

single experiment. Furthermore, the gas flux model indicated the importance of bubbles across this size range to gas flux; however this must be offset by the practical consideration that acoustic sources tend to cost a similar amount per octave (see above). Since the pulsation resonance of a bubble is approximately inversely proportional to its radius, this means that there must always be some large bubble size above which acoustic techniques cannot be used to probe for a resonance. Omission of large bubbles from the consideration of attenuation can lead to inaccuracies if they are present, since such large bubbles produce large geometrical scatter[53-55] (such that a key assumption in the inversion of measured attenuation to infer BSD is that the product of the largest bubble radius and highest acoustic wavenumber are much less than unity). This concern is offset by the fact that for the vast majority of bubble populations formed by breaking waves, at the hydrophone depths the number of bubbles falls off sharply with increasing bubble size[56].

Once the maximum and minimum frequencies had been chosen, the smaller the increment in frequency of pulses between these limits, the finer the resolution with which the BSD is estimated. However the experimenter is not free to choose many such frequencies, since ideally there should be stationarity within the bubble cloud, i.e., the population within the cloud should not change during time required to emit the pulse train. In reality, this is not possible with multiple pulse measurements, and a compromise was chosen such that a train of 14 pulses, each of 1 ms duration and an off-time between them of 20 ms, generated a pulse train of 274 ms (figure S2.1(b)). For the assumption of stationarity to hold, the bubble cloud would need not to change significantly over the 274 ms measurement. This would require, for the experimental setup used here, that the average velocity of the bubble cloud should not exceed 0.73 m s^{-1} for stationarity to hold. Whilst this may be exceeded directly beneath a breaking wave, for most of the time in a volume of sea at the location of the hydrophones, this velocity will not be reached. It was possible to check on the stationarity in the cloud over the pulse train at the measurement depths, because the pulse train was repeated every second, allowing the bubble count in one size bin to be compared with that in the same size bin 1 s later.

Equal frequency increments between the pulses in the train would generate a population that was densely sampled for small bubbles, but sparsely for the large ones, because of the approximately inverse relationship between pulsation resonance frequency and bubble radius. A constant bandwidth spacing was the aim, compromised somewhat by the need to operate within the most sensitive range of each of the transmit transducers. Table S2.1 shows the frequencies of the pulses, which are the resonant frequencies f_0 for bubbles having equilibrium radii R_0 as calculated using equation (S2.1), which is a modified[57] form of that given by Minnaert[58] to include the effect of surface tension (σ) and shear

viscosity (η):

$$f_0 = \frac{1}{2\pi R_0\sqrt{\rho_w}}\sqrt{3\kappa\left(p_0+\frac{2\sigma}{R_0}-p_v\right)-\frac{2\sigma}{R_0}+p_v-\frac{4\eta^2}{\rho_w R_0^2}} \quad (S2.1)$$

The value of σ can strongly depend on the surface agents in the seawater which collect on the wall of the bubble as it moves through the ocean.[57] It is recognized that measurement of the 'effective' surface tension on a bubble wall is difficult, and this will likely differ from the 'surface tension' measured on a sample of seawater using traditional techniques, and for this reason new methods to measure the 'effective' surface tension on a bubble wall are just being developed[59,60]. Here σ = 0.036 N m^{-1} is used for a bubble wall in seawater loaded with surface active agents, and it is recognized[59,60] that future work is required to measure the value pertaining on the relevant bubble walls *in situ*. Similar comments pertain, to a lesser extent, to the shear viscosity, here taken to be η =0.001 Pa.s. In equation (S2.1), κ is the polytropic index (which varies between unity and the ratio of the specific heat of the gas at constant pressure to that at constant volume, depending on whether conditions are isothermal, adiabatic or in between). It allows for the change in the stiffness of the gas[56] as reversible heat flow occurs across the bubble wall, but does not allow for any net thermal losses across the bubble wall[48]. Here p_0 is the static pressure that would exist in the liquid at the centre of the bubble if the bubble were not there, p_v is the vapour pressure the liquid, and ρ_w is the density of the liquid.

S2.2 Equipment

To recap and provide further detail on the instrumentation described earlier, the acoustic bubble measurements were autonomously performed alongside optical ones (not included here as the only affordable optical instrument was only a home-built prototype,[45,46,65,66] not a validated commercial system). The combination of optical and acoustical techniques is powerful since optical techniques are most effective at the large bubble extreme where the $f_0 \approx R_0^{-1}$ relationship of equation (2.1) makes the cost of acoustical sensors prohibitive (as described above), but that same relationship makes acoustical sensors very effective at mitigating the resolution and field-of-view limitations experienced by optical techniques with very small bubbles. In addition to these measurements of the bubble size distribution, an upward-looking sonar produced sonar imagery of the shape and depth of bubble clouds (not true images because they are based on the scatter of the beam as clouds advect through it[61-64]). As with the commercial optical system for the BSD, and the additional hydrophones for the eventual 3-hydrophone array,

the budget was not approved for an acoustical resonator system[47] that it was hoped would complement the other measures of BSD and provide cross checks for them.

The 3-hydrophone and prototype optical systems, and the upward-looking sonar bubble cloud profiler, were mounted upon an autonomous spar buoy constructed by the National Oceanography Centre (NOC), Southampton, which in turn held a range of equipment for making oceanographic measurements:[46] capacitance wave wires and accelerometers to measure surface elevation and wave breaking and downward-looking video and still digital cameras to obtain images of the sea surface. The deployment of this spar buoy was in turn part of a larger programme[65,66] aimed at investigating the transfer of mass, momentum and energy between the atmosphere and ocean.

The spar buoy is 11 metres long with approximately 2.5 metres protruding above the sea surface (see Figure S2.2). The spar buoy had a damping plate on the bottom of it which, as well as holding the batteries, allowed the buoy to ride up and down with the long period waves (i.e., swell) and yet not with the shorter period waves (i.e., breaking waves). This allowed capacitive wave wires (developed at NOC) mounted on the top section of the buoy to measure wave height and, from this, detect breaking waves (Fig. S2.3).[46,65,66]

S2.2.1 Power management and distribution

A total of three Deep Sea Batteries are mounted on the very bottom section of the buoy, two of which are used for the acoustic systems, the other is used for the wave wires, cameras and positioning equipment (Figure S2.2). The two voltages from the batteries were 12 V and 24 V. Outputs from Vicor DC-DC Converter Modules were connected to a distribution board from which the necessary voltages were supplied to each piece of equipment.

S2.2.2 Controlling the equipment

The buoy was to be free floating and autonomous, which required that a suitable control system had to be developed. At the heart of the system was a MagnumX 1000 Single Board Computer running Windows XP. This computer has an average power consumption of approximately 12 W as opposed to a typical desktop computer at the time, which would use upwards of 100 W. It was important to keep the power consumption as low as possible since energy would potentially be the limiting factor in determining the maximum length of time over which the acoustic system could acquire data. A low power unit will also produce far less heat, which aids the prevention of potentially damaging condensation forming inside the housing. Script files were written in Mathworks MATLAB and were then compiled to run as executable files upon Windows startup. These scripts controlled the timings of each of the measurements as well as generating the acoustic waveforms that were sent out into the water. The received waveforms were recorded straight onto a Seagate Momentus hard drive. These hard drives

are of the 2.5” form factor as opposed to the standard 3.5” form factor. Standard hard drives at the time of deployment would have had a high probability of failing under the shock and motion that they would experience on the buoy whereas the 2.5” form factor drives were far more resilient to the violent motion at the sea surface.

The data bandwidth required of the hard drive was large and therefore it was important to ensure that the 2.5” hard drive (which, by nature, is slower than a 3.5” hard drive) would be able to handle the volume of data coming in from the acquisition card. At any one time, the acquisition card could be acquiring data on 4 channels at a maximum sampling rate of 1 MHz. This gives a maximum bandwidth of [4 channels × 1 MSamples/s × 2 bytes/sample] = 8 MB/s. The manufacturer’s specification for the hard drive states a maximum sustained transfer rate of 44 MB/s, though independent tests[67] suggested that after sustained periods of data transfer the rate may drop to approximately 22 MB/s. Even with this drop in transfer rate, the hard drive can comfortably maintain the required bandwidth.

S2.2.3 Data acquisition

All acoustic waveforms were generated on the computer and therefore had to be converted from digital signals to analogue signals. The received waveforms would also need to be converted from analogue signals to digitals signals. These procedures were carried out by a National Instruments PCI-6110 DAQ card plugged into the computer. This is a multifunction data acquisition card allowing simultaneous sampling over multiple channels for both input and output data at high sampling rates (a sample rate of 1 MHz per channel was used throughout the experiments). The card’s two available analogue outputs were both used, one sending signals to the low frequency side of the amplifier, the other sending signals to the high frequency side of the amplifier.

Prototype home-built optical fibre sensors were added to the system to provide an additional measurement of the bubble size distributions. This meant the data acquisition cards maximum number of input channels (4) was exceeded. A multiplexing board was custom designed and built to fit onto the data acquisition card. Sending a digital signal from the acquisition card to the board would switch between two banks of 4 input channels, increasing the number of available channels to 8, although only 4 could be used simultaneously. The regime for taking data was as follows; the BSD was measured once a second for 9 seconds, then passive acoustic measurements and measurements with the optical fibres were taken. After two and a half minutes (the time taken for passive acoustics and optical fibre measurements and for writing files to the hard drive), the BSD was measured again (once a second for 9 seconds). This cycle continued until the charge in the batteries was depleted. The upward looking sonar was scheduled to run twice for 10 minutes each time. These measurements were made

approximately 4.5 and 10 hours into the measurement period.

S2.2.4 Power amplification

The design requirements for the power amplifiers were such that an off-the-shelf amplifier could not be used since no existing battery-powered amplifier at the time could operate over such a broad range of frequencies (2-200 kHz) at the required power levels. Therefore the amplifiers had to be custom designed. In bubbly environments, attenuation is high and therefore the transmit level from the transducers must be large. A source level of approximately 190 dB re 1 μPa at 1 metre was set as the design aspiration, which was achieved.

As part of the amplifier, matching circuits were incorporated for each of the transducers in order to flatten the frequency response of each transducer and extend the usable range.[68] This is necessary to mitigate against the amplitude and phase effects around resonances in the transducers. As with the other electronics, the power amplifiers were contained in an underwater housing, mounted on the buoy. Being in an enclosed space can cause overheating issues, but this was overcome by sinking the amplifier chips into one of the end caps of the housing, thus dissipating the heat into the water outside the housing, rather than into the air contained within the housing. The plan to fill the containers with dry nitrogen to avoid condensation issues was not possible to implement on ship because of resource issues.

S2.2.5 Transmit transducers

In order to cover the required frequency range (2-200 kHz), a total of three transducers were used. A Massa 137D transducer was used for the low frequencies. This transducer has a usable frequency range of approximately 2-11 kHz. A custom made piston transducer, supplied by Neptune Sonar, was used for the mid frequencies. Piston transducers are naturally directional and very efficient, which is ideal for this application. The transducer is resonant at 24 kHz and with the matching circuits mentioned in the previous section has a usable frequency range of approximately 15-30 kHz.

The third transducer, used for high frequencies, is another custom designed and built transducer. The transducer has previously been used to measure bubble populations in the laboratory[69,70] and the oceanic surf zone.[48,51,52] The transducer actually has three elements inside, each one covering a different frequency range. In this experiment however, only two of these elements were used giving a total frequency range of 30-200 kHz.

S2.2.6 Hydrophones

The design criterion for the hydrophone system was challenging. The hydrophones were to be mounted close to the sea surface, whereas the data

acquisition equipment was to be mounted in the housing at the bottom of the buoy. This poses a problem of signal-to-noise ratio as the signals need to travel long distances along the cables before they are recorded, increasing the possibility of electrical interference and cross talk on the signal. One solution to this problem would be to use hydrophones with built in pre-amplifiers which could boost the signals before they are transmitted along the cables, thus greatly increasing the signal-to-noise ratio. There is however a problem with this solution. The near-surface ocean can be a very turbulent environment and therefore the hydrophones could be damaged by debris. The hydrophones were also to be mounted on arms protruding from the buoy and could easily be knocked and damaged during deployment and recovery of the buoy from the ship. Therefore it would be very costly to replace a hydrophone and a pre-amplifier as opposed to just a hydrophone. The solution that was decided upon was to use hydrophones with short cables connected to a pressure housing containing pre-amplifiers mounted close to the hydrophones. A long cable ran from this housing to the main housing, carrying the already amplified signals. This provided a good signal-to-noise ratio whilst only having the hydrophones, and not the pre-amplifiers, in a vulnerable position. The hydrophones used were D140 hydrophones, with a usable frequency range of 1 - 200 kHz, supplied by Neptune Sonar who also supplied the pre-amplifiers. The pre-amplifiers were designed with a 1 kHz high pass filter to filter out any low frequency noise, such as ship noise. There is a link that can be made on the printed circuit board of the pre-amplifiers to increase the gain from 20 decibels to 40 decibels.

A single B200 hydrophone, also supplied by Neptune Sonar, was mounted on the same plate as the transmit transducers. This hydrophone was used as part of an inverted echo sounder system and measured surface reflections. The B200 is a high performance, low noise hydrophone with a specified frequency range of 10 Hz - 180 kHz.

S2.2.7 Other Equipment

All of the electronics needed to be mounted on the buoy, and therefore had to be contained in a waterproof housing. There is a large amount of equipment and electronics associated with the acoustic system and most conventional housings are too small. A custom designed housing was manufactured by Neptune Sonar. The housing is cylindrical in shape with an internal diameter of 286 mm and a length of 644 mm.

There is sensitive electronic equipment on the buoy and during a deployment the buoy can be knocked against the side of the ship. In order to avoid this kind of shock occurring whilst the equipment is running, a water switch is used in conjunction with relays so that the electronics only switch on when the buoy is in the water.

All the necessary clamps to mount equipment on the buoy were designed and manufactured by the Engineering Design and Manufacturing Centre at the University of Southampton.

S2.2.8 System refinements

The buoy was deployed on two separate sea trials on the RRS Discovery (Fig. S2.3). The first sea trial (Cruise D313, 6 November to 14 December 2006) took place in sheltered areas amongst the islands to the west of Scotland as the ship sheltered from storms. The plan was to use the first sea trial (which was always designed to test the equipment and procedures, rather than to take data) to identify refinements to the equipment that could be made prior to the second sea trial, which would take the data for this paper. A number of issues were highlighted. Deploying the buoy from the ship in anything other than calm water was discovered to be very difficult (Fig. S2.3). Yet if data is to be taken during rough weather, a workaround needs to be developed. Ideally, the buoy would be deployed in calm water a couple of days ahead of a storm. Data would be taken throughout the storm and then, when the weather had calmed, the buoy would be recovered. This would result in deployments of days rather than hours as it had been in the first sea trial. With a maximum battery life of approximately 12 hours, it was clear the acoustic system would need a form of timing device to delay system startup until the storm was overhead. As a solution, a Finder 86 series timer module was installed between the water switch and the startup relays inside the main pressure housing. The timer module could delay the system startup for up to 100 hours (approximately 4 days) which was more than sufficient. Another discovery was that with the pressure housing containing the computer and power amplifiers being so heavy, lifting it to and from, and mounting it on the buoy was not trivial. It was seen as the most involved process in preparing the buoy for each deployment. Downloading the data whilst the housing was still on the buoy would make things very much easier. So an extra bulkhead connector was fitted in one of the end caps. Inside the housing, the 8 pins on the connector were attached to an ethernet cable running to the onboard computer. Outside of the housing, an ethernet cable was spliced onto a cable designed to mate with the bulk-head connector. This allowed remote access to the onboard computer from a laptop, which simplified the data downloading process dramatically. Powering the computer on deck was not trivial however. Previously, power to the housing would be initiated by the water switch and it was easy to short the contacts on the switch to override this. However, with the timer module now in place, power would not be supplied to the housing (and therefore the onboard computer) until the timer had finished which was typically 50 hours. This would negate any time savings brought about by being able to access the data on the computer remotely. To solve this problem, a reed switch was installed on the inside of the end cap. When a magnet was held against the correct place on the end cap, the reed switch would be activated and bypass the timer module, thus supplying power instantly

to the computer.

Time synchronisation of the acoustic system and wave wire system was an important issue to resolve. It is something that had not been properly addressed for the first cruise. This made it difficult to synchronise breaking wave events occurring on the wave wire data and video data with the acoustic data. This problem was solved by synchronising both systems with the ships clocks (set to Greenwich Mean Time) before each deployment. This would allow synchronisation accuracy within one or two seconds. The ambiguity arises because of an inherent drift within the clock on the computer causing a small error over the duration of the deployment.

S2.3 Experiments

The results reported here were collected on a sea trial from 16th June to 18th July 2007 on the *RRS Discovery* (Fig. S2.3). The ship sailed from Falmouth and returned to Govan, Glasgow. The area of operation was in the North Atlantic, approximately 400 miles west of Portugal (Fig. S2.4(a)).

The data presented here was on the second deployment of the second sea trial. The successful deployment was made after almost two weeks of calm weather. A strong weather front was predicted heading towards the area of operation. The front was tracked and a prediction was made as to when it would reach the ship's position. The buoy was deployed at 17:31 GMT on 28th June 2007, one day before the weather front was predicted to arrive. The start delay timer was set to approximately 18 hours (the timer uses an analog dial with only zero and 100 on the scale making precise timings impossible). This actually turned out to be nearer 19 hours so the equipment turned on at approximately midday on the 29th June 2007.

The acoustic system took data for about 12 hours until the charge in the batteries was depleted. The weather conditions during the deployment were excellent (Fig. S2.4(b)). The number of breaking waves was the highest it had been for the sea trial. The buoy was recovered at 18:52 GMT on 1st July 2007. Upon recovery, the buoy was sitting noticeably lower in the water. This was later discovered to be caused by a leak in the optical fibre pre-amplifier housing. This housing was half full of water which significantly reduced the buoyancy of the buoy, causing it to sit lower in the water. The difference was approximately 0.15 metres.

With the previously experienced computer problems fixed, it was possible to run the inverted echo sounder and passive acoustic systems as well as the attenuation system. Only three hydrophones were used in the attenuation array, with the fourth channel of the DAQ card being used by the hydrophone for the inverted echo sounder. The three hydrophones used in the attenuation array were also used for the passive recordings. The calculated depths are included in Table

S2.1.

S2.3.1 Baseline Attenuation

When inverted acoustic attenuation measurements to obtain bubble size distributions, it is the excess attenuation due to bubbles that is of interest, i.e. the excess attenuation above a baseline level of attenuation between the hydrophones (with no bubbles present). The most reliable baseline is extracted from the sea trial data from the second deployment of D320 in the following manner. For each frequency and hydrophone, the measured sound pressure levels for the entire deployment are plotted, which produces a consistent band of values from which the baseline can be read (by eye) as the centre of the band. The band is typically in the order of 1 dB thick, which introduces an element of uncertainty in the baseline attenuation. The uncertainty can be calculated by taking the minimum and maximum value of the band and calculating the uncertainty (A_{uncert}) around an attenuation value using the equation:

$$A_{\mathrm{uncert}} = \frac{20}{L_{\mathrm{hydro}}}\left[\log_{10}\left(P_{\mathrm{m-rat}}\frac{P_{\mathrm{alt\_base,i+1}}}{P_{\mathrm{alt\_base,i}}}\right)\right], \tag{S2.2}$$

where L_{hydro} is the distance between two hydrophones, $P_{\mathrm{alt\_base,i}}$ and $P_{\mathrm{alt\_base,i+1}}$ are the alternative baselines (taken from the maximum and minimum of the thickness of the bands), and $P_{\mathrm{m-rat}}$ is the ratio of measured pressures. The uncertainties are worked through to the values shown in Table 1.

S2.3.2 Inverted echo sounder measurements

Inverted echo sounder (IES) measurements were made alongside the attenuation measurements in order to obtain a profile of the bubbles clouds as they were advected past the buoy. Narrowband pulses centred around 160 kHz were directed at the sea surface and volumetric backscatter strength was calculated (following the method of Trevorrow[64]) from the measured reflections. The echo sounder emits one pulse every five seconds for five minutes, then saves the file and repeats this procedure for another five minutes. Since the priority of previous testing has been to ensure the correct working of the attenuation system, and owing to limitations imposed by computer malfunctions, the successful deployment presented here was the first time the IES system has been used fully in the open ocean. This meant that only 10 minutes of useful data were obtained. Also, changes to the timing of the sea trial meant that it was not possible to calibrate the IES system in the same manner that was used in Trevorrow[64]. However, the method described below was used to calibrate the data. Volumetric backscatter strength can be calculated from bubble size distribution using the scattering cross-section[53]. Since bubble size distributions were measured either side of the IES dataset, these could be used to calculate the absolute volumetric

backscatter strength at the time of the IES measurements. This assumes that the bubble population as a whole does not change significantly over the measurement period (approximately 30 minutes), which is not an unreasonable assumption. A total of 45 BSDs (spanning approximately 10 minutes) either side of the IES dataset were used. From these measurements, the absolute volumetric backscatter strength at a depth of 2 metres was calculated to be -75.785 dB (three decimal places in practical measurements of acoustic signals fails to recognize that even a good instrument calibration has uncertainties of 1 dB or more, but these decimal places are re-stated here because they were carried through only to avoid rounding errors in the numerical code). The uncalibrated volumetric backscatter strength at a depth of 2 metres (as measured by the IES system) was 3.127 dB. A correction factor for the IES dataset could then be calculated from the difference between these values, i.e. 78.91 dB. Corrections for surface displacement were found by measuring the position of the surface reflection for each pulse recording. With the position of the surface known, it is possible to plot S_V with the surface flattened to give a profile of the sub-surface bubble clouds. Removing the surface displacements allows the calculation of time-averaged profiles. Figure 2.5 shows the variation in volumetric backscatter with depth and time, as measured by the IES system. This provides a useful insight into the vertical and horizontal variation of the sub-surface bubble clouds. Owing to limitations described above, the timing of the IES measurements did not correlate with a period of high bubble activity, and therefore the relationship between the IES results and attenuation measurement results is not as strong as it could have been. It is recommended that future measurements make attempts to alternate IES and attenuation measurements in a manner as to provide strong links between the two datasets.

References Supplementary Section 2

42 Coles, D.G.H. Measurements of sub-surface bubble populations and the modelling of air-sea gas flux. PhD thesis (University of Southampton, March 2010) (2010).

43 Leighton, T.G. & White, P.R., Quantification of undersea gas leaks from carbon capture and storage facilities, from pipelines and from methane seeps, by their acoustic emissions, *Proceedings of the Royal Society A*, **468**, 485-510 (2012) (doi: 10.1098/rspa.2011.0221).

44 Leighton, T.G., Baik, K. & Jiang, J., The use of acoustic inversion to estimate the bubble size distribution in pipelines, *Proceedings of the Royal Society A*, **468**, 2461-2484 (2012) (10.1098/rspa.2012.0053)

45 Coles, D. G. H., Hsueh, P-C. & Leighton, T.G. Acoustic and optical measurements of bubble populations in the Atlantic ocean and the modeling of gas transfer through these bubble clouds, *Proceedings of the*

Third International Conference on Underwater Acoustic Measurements, Technologies and Results, (Nafplion, Greece, 21-26 June 2009), 747-752 (2009).

46 Pascal, R.W., *et al.* A spar buoy for high-frequency wave measurements and detection of wave breaking in the open ocean, *Journal of Atmospheric and Oceanic Technology,* 28(4), 590-605 (2011).

47 Farmer, D.M., Vagle, S. & Booth, D. Reverberation effects in acoustical resonators used for bubble measurements, *J. Acoust. Soc. Am.* **118**, 2954 (2005) (doi: 10.1121/1.2047148).

48 Leighton, T. G., Meers, S. D. & White, P. R. Propagation through nonlinear time-dependent bubble clouds and the estimation of bubble populations from measured acoustic characteristics. *Proc. Roy. Soc. Lond. A* **460**, 2521–2550 (2004) (doi:10.1098/rspa.2004.1298).

49 Coles, D.G.H. & Leighton, T.G. Autonomous spar-buoy measurements of bubble populations under breaking waves in the Sea of the Hebrides, *Proceedings of the Second International Conference on Underwater Acoustic Measurements, Technologies and Results,* (Heraklion, Crete, Greece, 25-29 June), 543-548 (2007).

50 Clarke, J.W.L. & Leighton, T.G. A method for estimating time-dependent acoustic cross-sections of bubbles and bubble clouds prior to the steady state, *Journal of the Acoustical Society of America,* **107**(4), 1922-1929 (2000).

51 Leighton, T.G., *et al.* The Hurst Spit experiment: The characterization of bubbles in the surf zone using multiple acoustic techniques, In *Acoustical Oceanography* (Leighton, T.G., Heald, G.J. Griffiths, H. & Griffiths, G. editors), *Proceedings of the Institute of Acoustics*, **23**(2), 227-234 (2001).

52 Meers, S.D, *et al.* The importance of bubble ring-up and pulse length in estimating the bubble distribution from propagation measurements, In *Acoustical Oceanography* (Leighton, T.G., Heald, G.J. Griffiths, H. & Griffiths, G. editors), *Proceedings of the Institute of Acoustics*, **23**(2), 235-241 (2001).

53 Ainslie, M.A. & Leighton, T.G. Review of scattering and extinction cross-sections, damping factors, and resonance frequencies of a spherical gas bubble, *Journal of the Acoustical Society of America,* **130**(5), 3184-3208 (2011) (doi: 10.1121/1.3628321).

54 Leighton, T.G. From seas to surgeries, from babbling brooks to baby scans: The acoustics of gas bubbles in liquids, *International Journal of Modern Physics B,* **18**(25), 3267-314 (2004).

55 Ainslie, M.A. & Leighton, T.G. Near resonant bubble acoustic cross-section corrections, including examples from oceanography, volcanology, and biomedical ultrasound, *Journal of the Acoustical Society of America,* **126**(5), 2163-2175 (2009).

56 Leighton, T.G., Finfer, D.C., White, P.R., Chua, G-H. & Dix, J.K. Clutter suppression and classification using Twin Inverted Pulse Sonar (TWIPS), *Proc. Roy. Soc. A*, **466**, 3453-3478 (2010) (doi: 10.1098/rspa.2010.0154).

57 Leighton, T.G., *The Acoustic Bubble.* Academic Press, London, pp. 120, 306 (1994) (ISBN 0124419208).

58 Minnaert, M. On musical air-bubbles and sounds of running water. *Philosophical Magazine*, **16**, 235–248 (1933).

59 Leighton, T. G., Zhu, M. & Birkin, P. A technique to measure the real surface tension on a bubble wall, *Proceedings of the 2nd International Conference and Exhibition on Underwater Acoustics* (Rhodes, 22-27 June, 2014), pp. 169-176 (2014).

60 Leighton, T. G., Zhu, M. & Birkin, P. Measurement of the surface tension on a bubble wall, *Proceedings of the 7th Forum Acusticum* (7-12 September 2014, Krakow, Poland) paper SS20_1 (5 pages) (2014).

61 Thorpe, S., On the clouds of bubbles formed by breaking wind-waves in deep water, and their role in air-sea gas transfer. *Philos. Trans. R Soc London A* **304**, 155-210 (1982).

62 Osborn, T., Farmer, D. M., Vagle, S., Thorpe, S. A. & Cure, M. Measurements of bubble plumes and turbulence from a submarine, *Atmosphere-Ocean*, **30**(3), 419-440 (1992).

63 Dahl, P.H., Bubble clouds and their transport within the surf zone as measured with a distributed array of upward-looking sonars, *Journal of the Acoustical Society of America,* **109**(1), 133-142 (2001).

64 Trevorrow, M. V., Measurements of near-surface bubble plumes in the open ocean with implications for high-frequency sonar performance. *Journal of the Acoustical Society of America,* **114**, 2672–2684 (2003).

65 Brooks, I.M., *et al.* Physical Exchanges at the Air–Sea Interface: UK-SOLAS Field Measurements, (DOI: 10.1175/2008BAMS2578.1; with electronic supplement at DOI: 10.1175/2008BAMS2578.2), *Bulletin of the American Meteorological Society,* **90**, 629-644 (2009).

66 Brooks, I.M., *et al.* UK–SOLAS field measurements of Air–Sea Exchange: Instrumentation, electronic supplement (DOI:

10.1175/2008BAMS2578.2), *Bulletin of the American Meteorological Society*, **90**, ES9-ES16 (2009).

67 www.tomshardware.com (last viewed May 30 2011).

68 Doust, P.E. & Dix, J.F. The impact of improved transducer matching and equilisation techniques on the accuracy and validity of underwater measurements. In *Acoustical Oceanography* (Leighton, T.G., Heald, G.J. Griffiths, H. & Griffiths, G. editors), *Proceedings of the Institute of Acoustics*, **23**(2), 100–109 (2001).

69 Leighton, T. G., Finfer, D. C., Grover, E. J. & White, P. R. An acoustical hypothesis for the spiral bubble nets of humpback whales and the implications for whale feeding. *Acoustics Bulletin*, **22**(1), 17–21 (2007).

70 Leighton, T.G., Finfer, D.C. & White, P.R. Cavitation and cetacean, *Revista de Acustica*, **38**(3/4), 37-81 (2007).

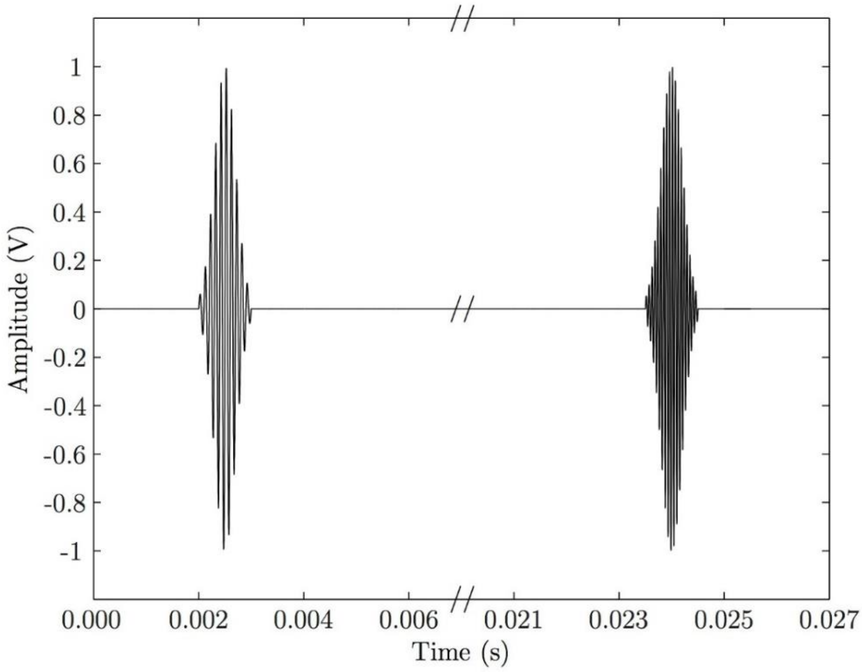

(a)

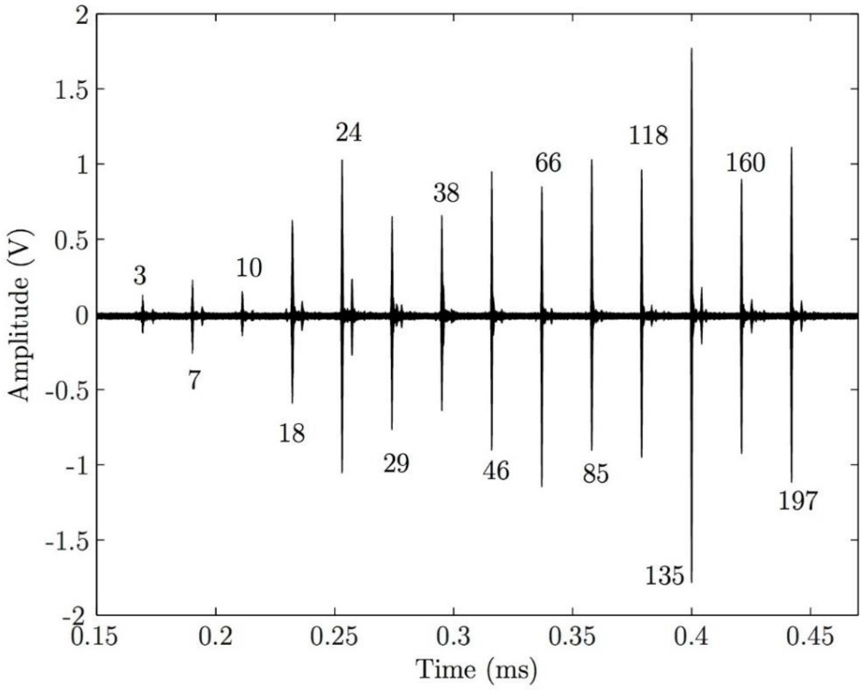

(b)

Figure S2.1 (a) Two of the concatenated pulses in the pulse train (as generated by the computer on board the spar buoy) with frequencies 10 kHz and 18 kHz. The pulse length and off-time are 1 ms and 20 ms respectively. The time axis is broken to remove the space between the pulses. (b) The 14 acoustic pulses as measured by the hydrophones on the sea trials, with frequencies labelled in kHz. This pulse train is repeated every second. The amplitude of each pulse respectively (in dB *re* 1 μPa) is 170, 175, 171, 184, 190, 186, 187, 189, 195, 193, 191, 198, 185 and 196.

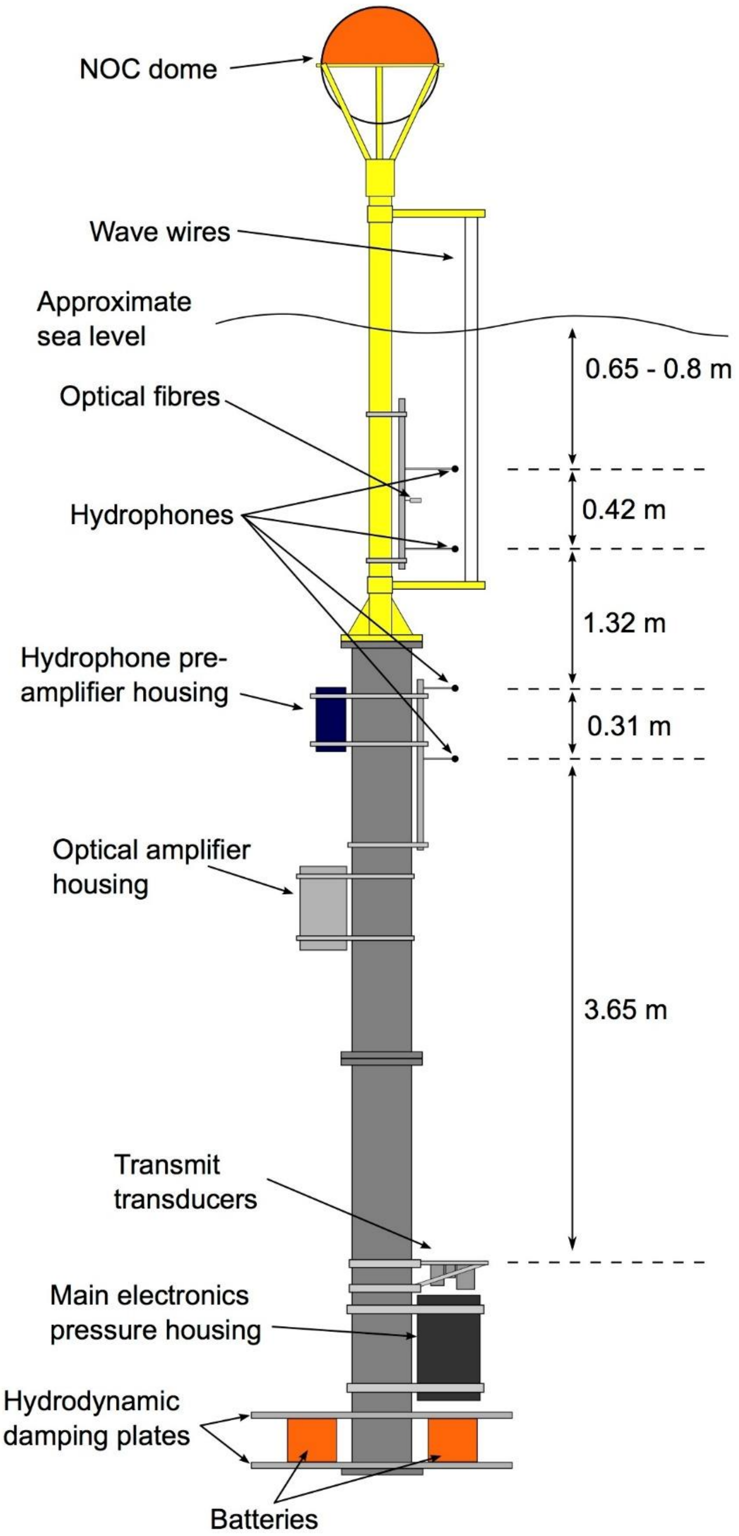

Figure S2.2 Schematic of the Spar Buoy (photographs of which can be seen in Figure 2 of the main paper, with movies in the electronic supplemental material).

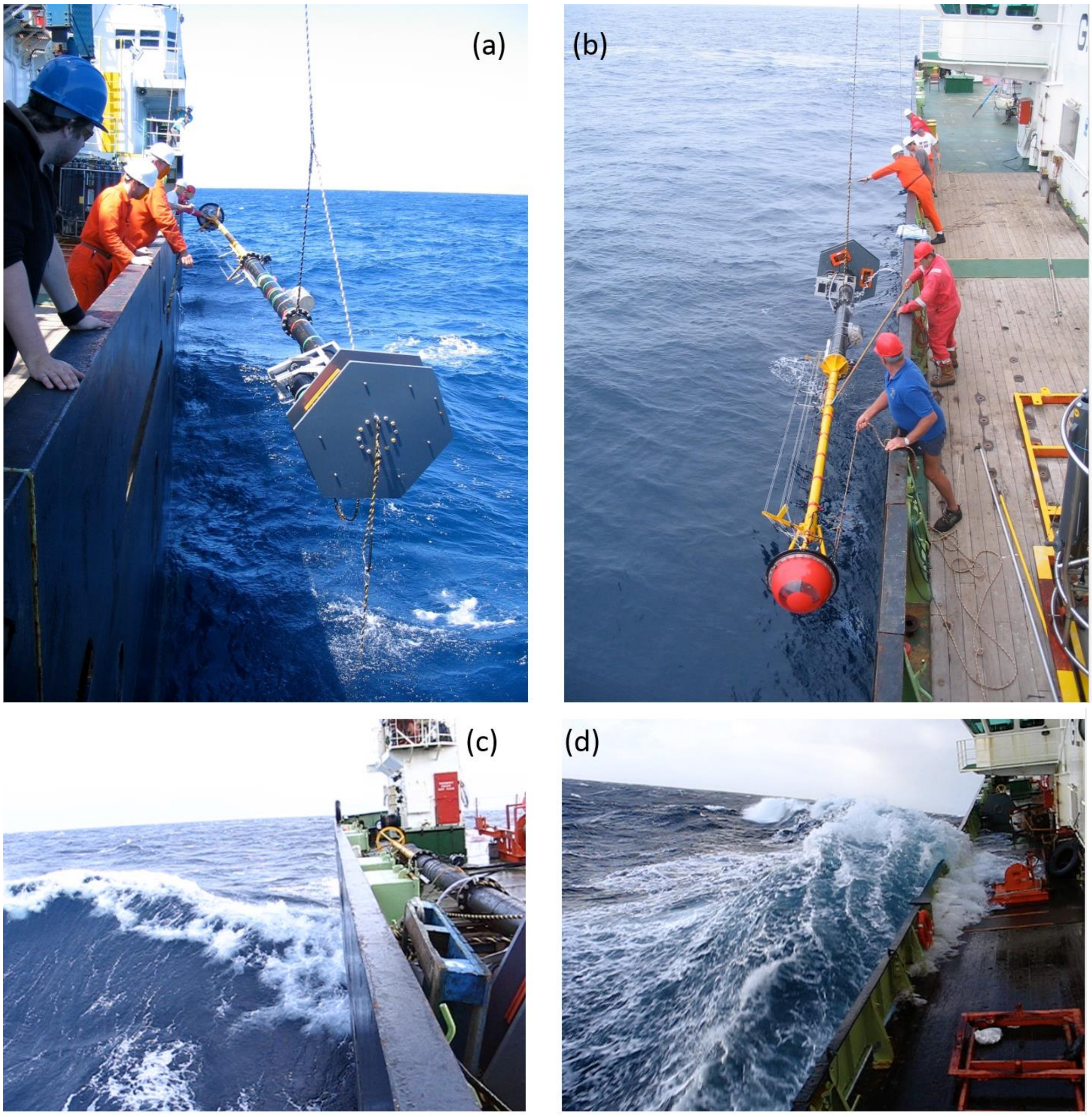

Figure S2.3 (a) The 11 metre long buoy being deployed from the *RRS Discovery*, seen from the base end, and (b) seen from the upper end. (c) Rough weather encountered during the voyage with the buoy seen stowed on deck, and (d) with waves topping deck level (videos illustrating the sea states encountered can be found in the supplementary material, entitled ‘Rolling ship’ and ‘Waves on deck’).

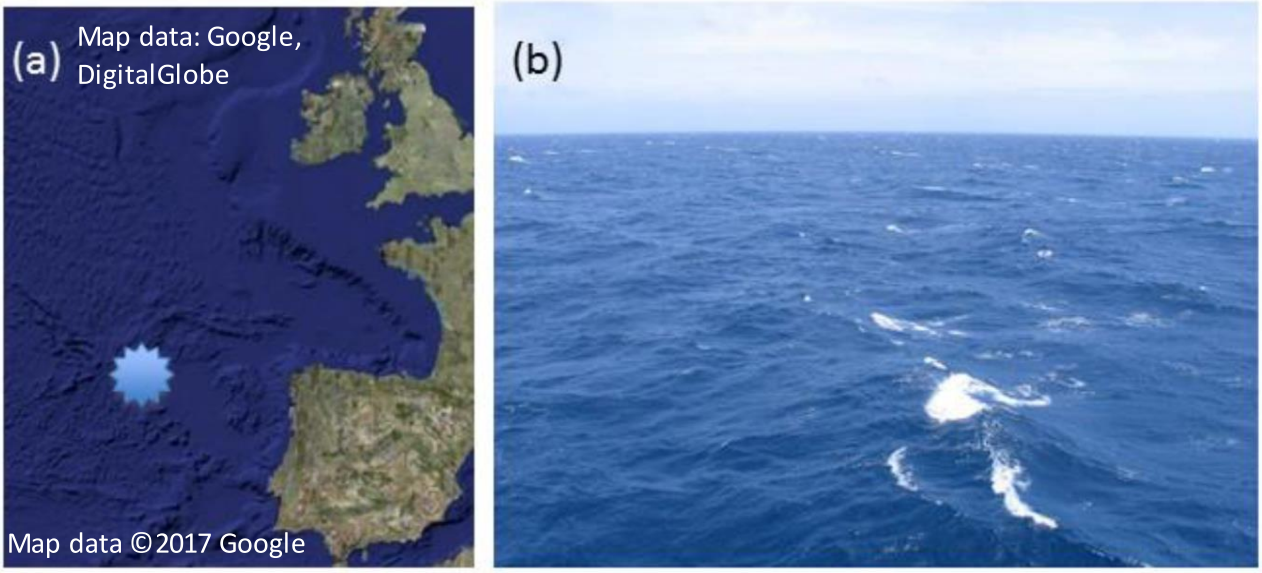

Figure S2.4 (a) The location where the data of this paper were taken. (b) Weather conditions in the time period that acoustic data was recorded on the second deployment of Cruise D320. The map element was created using Google Maps (version Google Maps API Version 2, https://www.google.co.uk/maps/).

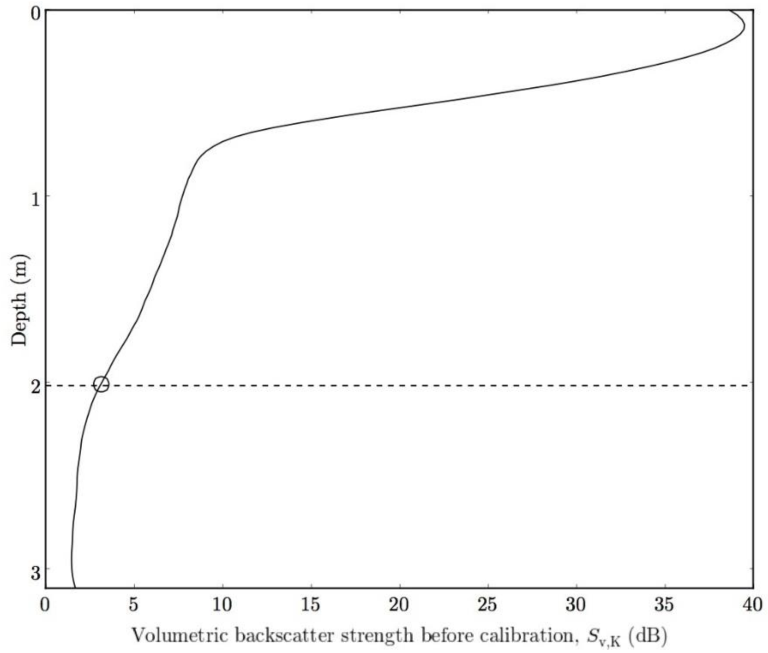

(a)

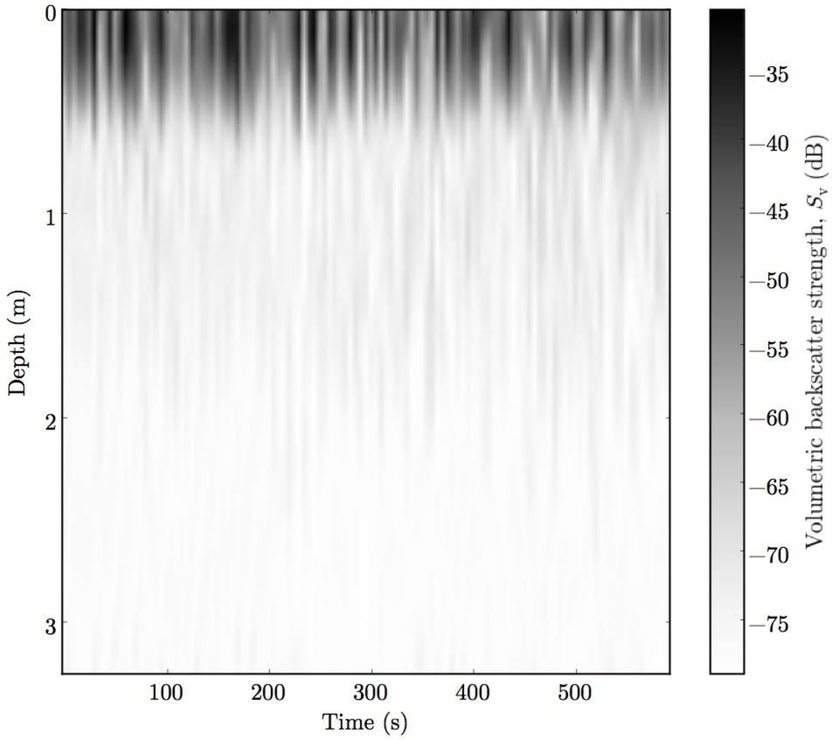

(b)

Figure S2.5: Volumetric backscatter strength as measured by the IES system inverted echo sounder system between 16:34 and 16:44 GMT on 29th June 2007. Panel (a) shows the mean vertical variation. The values are uncalibrated, and a depth of 2 metres is marked to show the value that is used in the comparison with the volumetric backscatter strength calculated from the BSDs measured either side. Panel (b) shows the final calibrated results and the variation in volumetric backscatter strength with time.

Tables

Pulse centre frequency, kHz	Resonant equilibrium bubble radius, μm
3	1141
7	486
10	339
18	187
25	134
29	115
38	88
46	72
66	50
85	39
118	27
135	24
160	20
197	16

Table S2.1: The frequencies used in the experiments, and the corresponding resonant radii calculated using equation S2.1 at a depth of 1.2 metres, assuming air bubbles in water and κ varying between κ = 1.15 for a radius of 16 μm and κ = 1.37 for a radius of 1141 μm as described in the literature[57].

Supplementary Section 3: Bubble-Cloud and Gas-Flux Modelling

This section describes the models used to interpret the experimental data on bubble size distributions. We explain how the measured data can be used with these models to produce meaningful results for gas exchange between the atmosphere and the ocean. Fuller details have been made available.[71]

As explained in Supplementary section S1, there are two contributions to gas flux between the atmosphere and the ocean: direct transfer and bubble-mediated transfer. Estimating bubble-mediated gas transfer through bubbles is not trivial. It is important to know the history of a bubble if the gas flux is to be calculated. This can be determined through the use of a model of the evolution of sub-surface bubble clouds. This is explained in more detail in the next section.

Historically, inputs to bubble models and parameters used have mostly been based on theoretical estimates.[72,73] They have also been limited by computational power. A novel aspect of this study is the use of actual measured data in the models.

S3.1 Bubble-cloud modelling

Thorpe[72] established a model for bubble cloud evolution based on equations for the dynamics of bubbles described by Garrettson.[74] The model took account of bubble rise speeds, changes in hydrostatic pressure, gas flux in and out of the bubbles (for oxygen and nitrogen), and turbulence in the water. The model produces sensible results with bigger bubbles remaining near the surface and smaller bubbles penetrating deeper because of their lower rise speeds and therefore their increased ability to be dragged down by turbulence (see Figure S3.1).

This model has a number of limitations. Firstly, it only describes bubbles composed of two gases, a mixture of nitrogen and oxygen. It cannot be modified simply to simulate more than two gases. Secondly, the model in this form does not include the effect of Langmuir circulation and the turbulence used in this model is constant with depth. Woolf and Thorpe[72] extended the original Thorpe model. They took a different approach to the calculation of molar content. The molar content of each individual gas was calculated based on exchange coefficients and partial pressures, from which the new bubble radii could be calculated. This approach was adopted for this study. The gases are nitrogen, oxygen, carbon dioxide and argon, and are the four gases with the highest volume-fraction in the composition of the atmosphere.

The model treats each bubble as an individual particle, whose motion and radius are changed and tracked throughout the model run.[75,76] Each model run is

broken down into small time steps and each bubble is subjected to a number of calculations (based on bubble dynamics and gas flux equations) at each time step. The theory behind these calculations is outlined in the following sections.

S3.1.1 Bubble dynamics equations

There are a number of key equations used in the model that affect bubble dynamics. The pressure inside each bubble can be calculated using:

$$p_{\mathrm{bub}} = p_{\mathrm{atm}} + \rho_{\mathrm{w}} g z + 2\sigma / R_0 \tag{S3.1}$$

where p_{atm} is the atmospheric pressure, $2\sigma / R_0$ is the excess pressure induced by surface tension (σ), and $\rho_{\mathrm{w}} g z$ is an approximate form[77] for the hydrostatic pressure.

Bubble rise speed is an important factor in the model and is different for hydrodynamically clean and dirty bubbles[78,79] (see Figure S3.2). A dirty bubble is a bubble covered in sufficient surface-active material for it to behave as a rigid body.[72] A clean bubble is completely free of surface-active material. The dynamic surface of a moving bubble will collect surface active agents such that the surface tension on a bubble wall might differ from that obtained from flat air/water interface (e.g. through a Langmuir trough measurement)[80]. As such the actual surface chemistry of an ocean bubble is one of the key unknowns, and currently very difficult to measure, although techniques are being developed to tackle this problem[81,82]. The model used here assumes that the bubbles are hydrodynamically dirty since that is the more typical behaviour in seawater.[83-86] The rise speed w_{b} of a dirty bubble is given as:[78]

$$w_{\mathrm{b}} = \left(\frac{2R_0^2 g}{9 v_{\mathrm{k}}} \right) \left(\sqrt{y^2 + 2y} - y \right) \tag{S3.2}$$

where v_k is the kinematic viscosity, and y is expressed as:

$$y = \frac{10.82 v_{\mathrm{k}}^2}{R_0^3 g}. \tag{S3.3}$$

S3.1.2 Gas exchange equations

Before the gas flux across the bubble wall can be calculated, a number of parameters must first be found. The Reynolds number of each bubble and the Peclet number and Nusselt number of each gas in each bubble must be calculated. The Reynolds number gives a measure of the ratio of viscous forces to inertial forces for the flow around the bubble[87] and is the same for each gas. It is given by:[88]

$$\mathrm{Re} = \frac{2R_0 w_\mathrm{b}}{\nu_\mathrm{k}}. \qquad \text{(S3.4)}$$

The Peclet number is ratio of the thermal energy convected to the fluid to the thermal energy conducted within the fluid. It differs for each gas and is given by:

$$\mathrm{Pe} = \frac{R_0 w_\mathrm{b}}{D_\mathrm{mol}}, \qquad \text{(S3.5)}$$

where w_b is the bubble rise speed as defined in Equation S3.2 and D_mol is the molecular diffusivity of each dissolved gas in water (values can be found in Table S3.1)[89,90]. The Nusselt number for each bubble depends on the Reynolds number and Peclet number for each bubble. The following formulae were used (for hydrodynamically dirty bubbles),

$$\mathrm{Nu} = 1.292\,\mathrm{Pe}^{1/9} \qquad \mathrm{Pe} < 24.17$$

$$\mathrm{Nu} = (2/\pi)\mathrm{Pe}^{1/3} \qquad \mathrm{Pe} > 24.17\ \&\ \mathrm{Re} < 8.017 \qquad \text{(S3.6)}$$

$$\mathrm{Nu} = 0.45\,\mathrm{Re}^{1/6}\mathrm{Pe}^{1/3} \qquad \mathrm{Re} > 8.017.$$

As described in Supplementary Section S1, a net flux across an air-water interface is driven by a concentration difference between the interface and the concentration in the water far beyond. The concentration of each gas at the surface of a bubble is given by:

$$C_\mathrm{w,i} = S_\mathrm{i} f_\mathrm{frac,i} p_\mathrm{atm} \varphi_\mathrm{sat,i}, \qquad \text{(S3.7)}$$

where i denotes the particular gas that is under consideration, S is the solubility, f_frac is the fraction in dry air and φ_sat is the saturation of each gas in the water. The concentration of each gas in a bubble is given by:

$$C_\mathrm{bub,i} = S_\mathrm{i} p_\mathrm{p,i}, \qquad \text{(S3.8)}$$

where p_p is the partial pressure of gas in the bubble. The flux of gas into the bubble can now be calculated as:

$$J_\mathrm{bub,i} = -4\pi R_0 D_\mathrm{mol,i} \mathrm{Nu}_\mathrm{i} (C_\mathrm{bub,i} - C_\mathrm{w,i}). \qquad \text{(S3.9)}$$

S3.1.3 Water motion equations

For the calculations in this paper, the approximation is made that that any motion of the water surrounding a bubble causes the bubble to move with the bubbles' terminal rise velocity simply superposed on the motion of the water (other accelerations of the bubble are neglected). The main factors affecting

fluid motion are:

- The initial jet of water from a breaking wave.
- Turbulence in the water.
- Langmuir circulation.

According to Woolf and Thorpe,[72] the initial jet of a breaking wave was modelled by using a triangular source distribution of bubbles of the form

$$\partial n / (\partial R_0 \partial z) = a(R_0)(1-2z) \qquad 0<z<0.5 \text{ m} \qquad \text{(S3.10)}$$

$$= 0 \qquad z<0, \qquad z>0.5 \text{ m}$$

where $a(R_0)$ describes the size distribution. This approach does not attempt to model actual bubble motion resulting from the jet. The approach taken here is different. Each bubble is given an initial velocity that linearly reduces to zero as time progresses. The time for the velocity to reach zero should be approximately 1 second since Deane and Stokes[91] found through experimentation that the active phase of a breaking wave in which the primary plume formation occurs is on average 1 second in duration. Initial velocity induced by the jet was varied in the parameter study but initially was based on relevant literature of plunging jets[17,18] described in more detail in section S3.3.3.

Turbulence is modelled through use of a Monte Carlo model. In each time step, each bubble is assigned a random motion in three dimensions. In the horizontal dimensions, each bubble is moved by a distance

$$\Delta x = v_0 \sin(\theta_x), \qquad \text{(S3.11)}$$

and

$$\Delta y = v_0 \sin(\theta_y), \qquad \text{(S3.12)}$$

where θ is chosen at random and v_0 is a prescribed velocity related to the turbulent diffusion coefficient and is given by:

$$v_0 = \sqrt{6K_v \Delta t}, \qquad \text{(S3.13)}$$

where K_v is the turbulent diffusion coefficient and Δt is the time step. The vertical movement is given by:

$$\Delta z = v_0 \sin(\theta_z). \qquad \text{(S3.14)}$$

The depth-dependent turbulent coefficient is given by:

$$K_v^z = 0.4 K_v z \,. \qquad (S3.15)$$

For depths less than 0.1 metres, K_v^z is set at the value for 0.1 metres. This technique is based on previous theoretical proposals[92,93] but adjusted here empirically in an attempt to mimic observed distributions. Quantitative values for K_v are outlined in section S3.3.3.

Langmuir circulation is a wind and wave driven phenomenon in which an array of vortices of alternating signs exists below the ocean surface with horizontal axes parallel with the wind direction.[94] It is beyond the scope of this investigation to model the effects of Langmuir circulation perfectly, but the realism of the gas flux model can be improved by including a simplified model for Langmuir circulation[72]. The effect is therefore modelled as a fixed field of cells defined by the stream function.

$$\Psi_L = \Psi_0 \sin k_L z \sin l_L y \,, \qquad (S3.16)$$

where k_L and l_L are the vertical and horizontal repetition lengths of the cells. The function Ψ_0 is given by:

$$\Psi_0 = V_L / l_L \,, \qquad (S3.17)$$

where V_L is the maximum downward or upward velocity (a user-defined parameter). The streamline function defines flow velocities (v in the horizontal and w in the vertical direction) at every point as:

$$v = \partial \Psi_L / \partial z \,, \qquad (S3.18)$$

$$w = -\partial \Psi_L / \partial y \,. \qquad (S3.19)$$

These Eulerian flow velocities can be used within the Lagrangian model, but the substitution requires short time steps to avoid a substantial error. Quantitative estimates for these parameters are detailed in section S3.3.1.

S3.1.4 Input bubble population

There is currently insufficient data to confirm a suitable input bubble size distribution when a wave breaks, and so an estimate is made for the model. Deane and Stokes[91] made some pioneering measurements of breaking waves and the initial bubble size distribution they produce. They found that the spectrum exhibits two power-law scales, with an increase of slope for bubbles larger than 1 mm in radius. This is caused by larger bubbles being affected by turbulent fragmentation and smaller bubbles being created by jet and drop impacts on the wave face. The spectrum can be seen in Figure S3.3. For bubbles smaller than 1 mm, the number of bubbles varies as $R_0^{-10/3}$ and for bubbles larger than 1 mm,

this varies as $R_0^{-3/2}$. An upper cut-off of 10 mm was chosen as this is approximately the point at which there is less than 1 bubble m^{-3}. A lower radius cut-off of 10 μm was chosen since this was deemed sufficiently low that very few bubbles would initially be formed at this radius. Testing of the model using only the outer few radii confirmed that these were viable assumptions since these tests resulted in no bubbles at the measurement depths.

The input population is spread randomly over a breaking area to simulate a breaking wave more realistically. It is also possible to change the depth at which the bubbles are inserted into the model in order to simulate surface disruption that may occur during a breaking event.

S3.1.5 Modelling process

With the core equations in place, it is possible to set about modelling the evolution of the bubble cloud. The model is implemented using MATLAB®. Each bubble is modelled individually and each property of the bubbles is stored in its own vector, so that the first index in each vector corresponds to the properties of the first bubble, the second index to the second bubble, the third index to the third bubble and so on. With the bubble properties in vectors, it is then easy to perform vector calculations for all the bubbles at the same time whilst maintaining individual bubble properties.

Each model run is split up into small time steps and for each time step the following calculations are made:

- New bubbles are added to the model, if specified.
- Bubble rise speeds are calculated.
- Reynolds numbers, Peclet numbers and Nusselt numbers are calculated.
- The concentration difference and the gas flux are calculated.
- New bubble radii are calculated based on the new total number of moles in each bubble.
- Bubbles that have dissolved are removed from the simulation.
- Langmuir circulation effects, the turbulent motion of the surrounding water and the rise velocities are applied to the bubbles.
- Bubbles that have surfaced are removed from the simulation.
- New partial pressures are calculated.

Depending on the type of model run, this process is repeated until all the bubbles have either surfaced or dissolved (in the case of a single initial injection), or the

bubble cloud has reached a steady state (in the case of repeated input of bubbles). At each step, the gas flux from each bubble is logged for reference once the model has finished.

If the method were to be extended to bubbles introduced into the water column from the sea bed, as opposed to the atmosphere/ocean interface (for example to investigate methane seeps, leaks from carbon capture and storage facilities etc.)[95,96] then there would be differences in injection dynamics, circulation, currents and turbulence at depth. Furthermore, all bubbles would reach the deepest depth, and consideration would need to be taken of the role of any gas hydrates present.

S3.2 Computational power

Computational power has vastly increased since Woolf and Thorpe[72] originally implemented their model. This section highlights some of the key aspects of progress possible with increased computational power.

S3.2.1 Model time step

With the computational power available to them, Woolf and Thorpe[72] were limited to a time step of 1 second. This is problematic because of the equilibration time of the gases. In each time step, the concentration of a gas in a bubble exponentially approaches an equilibrium value with the surrounding water. The time taken for this equilibrium value to be reached is the equilibration time. Figure S3.4 shows the time taken for each gas to reach equilibrium for a range of bubble radii. It can be seen that if the time step is 1 second, carbon dioxide will equilibriate in bubbles smaller than approximately 250 µm. Therefore, Woolf and Thorpe[72] assumed that carbon dioxide would equilibriate with each time step and they set the value to that of the surrounding water. Whilst this is not an unreasonable approximation, it is not entirely accurate, and with greatly increased computational power it is possible to reduce the time step to a value lower than the equilibration time of carbon dioxide in small bubbles.

S3.2.2 Number of bubbles in input population

As mentioned in section S3.1.5, each bubble is treated as a separate particle. If the number of bubbles in the model is increased, so is the length of the property vectors and in turn the computational load is increased. With the computational power available today, it is possible to model millions of bubbles. Model runs typically took between 2 hours and 2 days to complete depending on the time step and number of bubbles chosen. Clearly, if many runs of the model are to be completed, minimizing the time taken for the model to finish whilst still maintaining an acceptable level of accuracy is important. The total number of bubbles between 10 µm and 10 mm in the population shown in Figure S3.3 is

approximately 19 million bubbles. Now take for example a reduction in this number by a factor of 100. This results in an input population of approximately 200000 bubbles and can be seen in Figure S3.5. When the model has finished, any resulting data is scaled up accordingly (in this case by a factor of 100). However, a problem arises for larger bubbles with a total number of less than 1. Since each bubble in the model is represented by an index, there can only be whole numbers of any bubble size. So for bubbles below the line on Figure S3.5, the number must be rounded to either 1 or 0. This causes inaccuracies in the number of bubbles input to the model and the resulting bubble size distributions are affected. Whilst there is little impact on the amplitude of the distributions, quantization of the data occurs for more bubble radii as the input population is increasingly scaled down. Figure S3.6 shows the effect of these inaccuracies. For many of the runs undertaken in the parameter study (see section S3.3) it was deemed acceptable to use a reduced input population in order to reduce processing time. However, for the final results, the criterion for the number of bubbles used in the input was based on the required accuracy of the output, and bubble numbers were allowed to increase (with a consequent increase in computational time) until the required accuracy was achieved.

S3.3 Parameter study

Once the model for bubble cloud evolution and bubble-mediated gas flux had been developed, it became important to relate it to the data measured in the Atlantic Ocean (see Supplementary Section S2). This is achieved by trying to infer as accurately as possible the oceanic conditions that the measurement equipment experienced in the experiments, and then apply this to the model. Then once the model has finished, the bubble size distribution is taken and compared to that of the measured data.

Many, though not all, of the parameters in the model can be set at measured values. The following section explains how some of these parameters were calculated.

S3.3.1 Ancillary data applied to the model

The spar buoy was not the only item of equipment being used as part of the D320 cruise. Many other measurements were being taken by other investigators,[97,98] and these provided ancillary data for that cruise that could be used to reduce uncertainty in the modelling reported in this paper. These measurements can be used to estimate some of the parameters in the model.

An autonomous atmospheric measuring system, AutoFlux[99], was installed on the ship. This system measures water temperature and the mean value (17°C) from the day of measurements can be used in the model. For want of temperature measurements at the location of the buoy, for the modelling it was assumed that the temperature of the water by the ship is approximately equal to

the temperature of the water surrounding the buoy. A similarity in meteorological conditions is validated further by comparing wind speeds[100] measured at the ship and at another buoy close to the spar buoy (see Figure S3.7). The wind speeds can be used to estimate the scales for the Langmuir cells. Thorpe *et al.*[101] give the horizontal distance between Langmuir cells as:

$$l_{\mathrm{L}} = 0.47W_{10} + 9.87, \tag{S3.20}$$

where W_{10} is the wind speed corrected to an elevation of 10 metres. Using a mean value for wind speed of 14 m s^{-1}, the horizontal spacing for the Langmuir cells is approximately 16 metres. The depth of the Langmuir cells is generally agreed to be approximately half the length of the horizontal spacing[102,103], therefore a value of 8 metres is used.

It is important to decide the number of seconds to use between inputs of bubbles in the model. Data from the downward looking cameras mounted in the dome on top of the spar buoy can be used to assess the frequency of breaking waves in the measurement period. This is not an ideal approach since the cameras only operated during daylight hours and therefore switched off at 18:00 GMT, just as the sea state was building, and before many of the bubble populations were measured. However, it is possible to infer an estimate of the breaking frequency using this data. Figure S3.8 shows an example of a breaking wave caught on camera. Video data throughout the day was analyzed to find the frequency of breaking waves. The results are shown in Figure S3.9. The figure shows the time between breaking events decreasing as time progresses. It has been shown that whitecapping is greater in developed seas than in developing seas,[104] which suggests that the breaking frequency continued to increase. The increasing wind speed is shown in Figure S3.7 and the steadily increasing sea state is shown in Figure S3.10. It is important to take into account the fact that only waves breaking within the field of vision of the camera could be recorded and shown in Figure S3.9. Throughout the analysis, many waves could be heard breaking nearby and these would have affected the bubble populations surrounding the buoy. Also, the bubble populations shown here would likely have been the result of one or two large waves breaking within several seconds of each other. With all of these factors in mind, the time between breaking events was initially set to 10 seconds, with values around this number being used in the parameter study.

S3.3.2 Standard-value constants

Some of the values used in the model are shown in Table S3.1. Additional parameters that use standard values are shown in Table S3.2.

S3.3.3 Unknown parameters

As described above, many of the model parameters are fixed to specific values.

Another 6 parameters may be varied within reason in order to obtain the best fit between the measured bubble populations and the populations taken at the end of a model run. These 6 parameters are:

1. Turbulent diffusion coefficient
2. The maximum upward/downward velocity from the Langmuir circulation
3. The initial input jet velocity
4. The time for the jet velocity to reach zero after injection
5. The insertion depth for the bubbles
6. Time between breaking waves

With this many parameters to vary, and the time it takes for a standard model run (between 20 and 40 hours), it simply was not possible to run a standard automated parameter study. If each parameter is given 10 values to vary between, approximately 30 million hours of computational time (at the rates available to the authors) would be required. Therefore a manual approach was taken to find the best fit with the parameters. Each parameter was varied around a starting value while the others were held constant in order to find the effect of each parameter on the resulting population. Sensible starting values for the maximum velocity from the Langmuir circulation (15 cm s^{-1}) and the turbulent diffusion coefficient (1×10^{-2} m^2 s^{-1}) could be taken from Woolf and Thorpe.[72] Deane and Stokes[91] show the active phase of a breaking wave, where primary plume formation occurs, to last for approximately 1 second and therefore this was set as the starting value for the time for the jet velocity to reach zero. A study of some relevant literature on plunging jets[105,106] reveals an inception velocity for turbulent jets of approximately 0.8 m s^{-1} and upwards. A starting value of 1 m s^{-1} was therefore used for the initial jet velocity.

With initial values set, an extensive study was undertaken, making use of the university computer cluster, Iridis. Iridis has over 1000 processor cores and allows many jobs to be run simultaneously. Without this, the parameter study would not have been completed. Each time a set of parameters was tested, the model would be run until the bubble cloud had reached steady state. This is when the number of bubbles in the cloud does not significantly change with each subsequent input of bubbles (see Figure S3.11). Once this had been reached, bubble size distributions were taken at depths corresponding to the measured data, i.e., 1.15 metres and 2 metres. These BSDs were directly compared with the measured bubble populations.

The final set of parameters that produced a good fit for the bubble size distributions at both depths are reported in Table S3.3. The main results are

reported in the main paper with some extra detail explained in the following section.

S3.4 Gas flux model results and analysis

Simulations were made both of individual breaking wave events (an example is shown in Figure S3.12) and more extended domains (an example of which is shown in Figure S3.13). These simulations were used to optimize the parameter values. The following sections and the main paper focus on time-averaged results using the final parameter values.

S3.4.1 Bubble size distributions (BSDs)

As shown in Figure 4 of the main paper, the simulated BSDs and the measurements show very good agreement at both depths for bubbles with radii between 16 and 187 μm. The agreement is less convincing for the larger bubbles. The discrepancy may be due to the number of the cycles in the low frequency acoustic pulses being too few (see section S2.1.1), or an unknown error in the model or measurement. It is also interesting to note the location of the peak in the bubble size distribution, at approximately 20 μm, agrees well with previous open ocean measurements.[107,108]

S3.4.2 Parameter Sensitivity, Uncertainty Analysis and Variability

Three key sources of uncertainty or variability are identified in the study:

- An uncertainty due to an inexact baseline (the acoustic attenuation without bubbles)
- Uncertainty in parameter values and the consequent sensitivity of the simulated BSDs
- Apparent variability during the measurement period (rising bubble numbers as the sea state rose).

All of these features are mentioned in the main paper (see especially Figure 6 on variation with sea state), therefore only a few additional details are provided here. There is uncertainty in the bubble size distributions arising from the baseline measurement. The baseline measurement is the measurement taken at sea with no bubbles present. It is necessary since the inversion of acoustic attenuations to bubble distribution requires the additional attenuation due to the presence of the bubbles. It is possible to follow this uncertainty through and assess the impact on the gas transfer coefficients. In order to do this, the best fit for the minimum and maximum bounds in the bubble size distributions must be found. Repeating the full procedure found in section S3.3 would take too much time and therefore a simplification is undertaken. By varying the number of bubbles in the input population for the computer model, the resulting bubble size

distributions can be scaled up and down. Having run the model for a range of input populations, the best fit is taken for the minimum and maximum bounds in the measured results. Figure S3.14 shows the best fit found in each case. The resulting scaling of the results is followed through in section S3.4.3. It was found that a scaling by 64% produced the best fit for the minimum bubble size distributions and a scaling by 144% produced the best results for the maximum bubble size distributions.

Since a number of parameters were initially unknown, we present the sensitivity of the model to parameter values, in order to illustrate that the modelled bubble size distributions are sensitive to each of these parameters. Figure S3.15 shows the change in the bubble size distribution when the "unknown" parameters are changed. Perturbation values were chosen as the minimum and maximum values used in the parameter study (see section S3.3.3). The change is shown at 1.15 m depth for each parameter except for the change in the breaking jet velocity since this had a significant effect on the relative magnitudes of the bubble size distributions at 1.15 and 2 metres depth. The turbulent diffusion coefficient is not shown, as the bubble size distributions were fairly insensitive to this variable. Apparently the Langmuir circulation plays a greater role in the distribution of the bubbles. As can be seen, the model output is sensitive to each parameter. Therefore, if similar experiments are to be conducted in the future, effort should be made to incorporate measurements of these parameters where possible. It may also be advisable to examine the method of modelling the initial jet from the breaking wave. At the same time, it is apparent that the measured bubble size distributions are effective in quite precisely fixing individual parameter values (however some pairs of parameter values may counteract in the bubble size distributions).

S3.4.3 Inference of gas flux

Formulae for gas transfer across a broken sea surface were discussed in section S1. We recall that the following equation is proposed for the gas flux mediated by bubbles:

$$F_b = K_b((1+\delta)C_a - C_w), \qquad \text{(S3.21)}$$

In fact, it is not obvious that an equation of this form is appropriate, though Woolf and Thorpe[72] found it was satisfactory for previous model calculations. The equation implies a linear dependence on dissolved gas supersaturation. It is possible to test whether the equation is appropriate by running the current model for a number of gas supersaturations, in which case the linear dependence can be tested. Further, where a linear dependence is demonstrated the parameters can be inferred from the "slope" and "x intercept" of the fitted straight line.

The model is run using the set of parameters decided earlier. However, instead of running the model with a continuous input of bubbles until the cloud reaches

a steady state, only a single input of bubbles is used and the model runs until all the bubbles have either surfaced or dissolved. The continuous input method mimics the observed conditions and serves to find the optimum parameters. The single input method then uses these parameters and gives the total gas flux associated with a specific input of bubbles. The dependence on saturation is discovered through repetition of the process for several saturations of each gas. Figure 5 of the main paper shows the results obtained using this method. The figure shows the net flux of gas (nitrogen, oxygen and carbon dioxide) out of the bubbles and into the ocean from an input of bubbles as that in Figure S3.3. Each time, the saturation of the gas in question is varied and the resulting flux of that gas is plotted. The saturation is related to the concentration of a gas in the water through the equation $C_{\mathrm{w}} = Sp_{\mathrm{p,w}}\phi_{\mathrm{sat}}$ (see Equation S3.7), where $p_{\mathrm{p,w}}$ is the partial pressure of a gas in water.

This figure shows the linear dependence of gas flux on saturation. Therefore, the applicability of Equation S1.7 is confirmed for all four gases.

Data availability statement: The University of Southampton data repository will issue a doi and web link to the archives of the data and computer code if paper is accepted (the policy is not to issue these before acceptance). The doi and link for the data can be found in the main paper.

References Supplementary Section 3

71 Coles, D.G.H. Measurements of sub-surface bubble populations and the modelling of air-sea gas flux. PhD thesis (University of Southampton, March 2010) (2010).

72 Thorpe, S.A. A model of the turbulent diffusion of bubbles below the sea-surface. *J. Physical Oceanography*, **14**(5), 841–854 (1984).

73 Woolf, D. K. & Thorpe, S. A. Bubbles and the air-sea exchange of gases in near-saturation conditions. *J. Mar. Res.* **49**, 435-466 (1991).

74 Garrettson, G. A. Bubble transport-theory with application to upper ocean. *J. Fluid Mechanics*, **59**,187–206 (1973).

75 Leighton, T.G. From seas to surgeries, from babbling brooks to baby scans: The acoustics of gas bubbles in liquids. *International Journal of Modern Physics B*, **18**(25), 3267-314 (2004).

76 Leighton, T.G., Meers, S.D. & White, P.R. Propagation through nonlinear time-dependent bubble clouds and the estimation of bubble populations from measured acoustic characteristics. *Proc. R. Soc. Lond. A*, **460**(2049), 2521-2550 (2004).

77 Leighton, T. G., Finfer, D. C. & White, P. R. The problems with acoustics on a small planet. *Icarus* **193**(2) 649–652 (2008).

78 Thorpe, S. A. On the clouds of bubbles formed by breaking wind-waves in deep water, and their role in air-sea gas transfer. *Philosophical Transactions of the Royal Society of London A (Mathematical and Physical Sciences),* **304**(1483), 155–210 (1982).

79 Levich, V. G. *Physico-Chemical Hydrodynamics*. Englewood Cliffs, Prentice Hall, NY (1962).

80 Maksimov, A. O. & Leighton, T. G. Pattern formation on the surface of a bubble driven by an acoustic field. *Proc. R. Soc. Lond. A,* 4**68**, 57-75 (2012) (doi: 10.1098/rspa.2011.0366).

81 Leighton, T. G., Zhu, M. and Birkin, P. A technique to measure the real surface tension on a bubble wall. *Proceedings of the 2nd International Conference and Exhibition on Underwater Acoustics* (Rhodes, 22-27 June, 2014), pp. 169-176 (2014).

82 Leighton, T. G., Zhu, M. and Birkin, P. Measurement of the surface tension on a bubble wall. *Proceedings of the 7th Forum Acusticum* (7-12 September 2014, Krakow, Poland) paper SS20_1 (5 pages) (2014).

83 Detsch, R.M. Dissolution of 100-1000 μm diameter air bubbles in reagent grade water and seawater. *J. Geophysical Research*, **95**(C6), 9765–9773 (1990) (doi: 10.1029/JC095iC06p09765).

84 Detsch, R.M. Small air bubbles in reagent grade water and seawater 1. Rise velocities of 20 to 1000 μm diameter bubbles. *J. Geophysical Research,* 96(C5), 8901–8906 (1991) (doi: 10.1029/91JC00484).

85 Harris, I. A. & Detsch, R. M. Small air bubbles in reagent grade water and seawater: 2. Dissolution of 20-500 μm diameter bubbles at atmospheric pressure. *J. Geophysical Research* **96**(C5), 8907-8910 (1991).

86 Lozano, M. M., Talu, E. & Longo, M. L. Dissolution of microbubbles generated in seawater obtained offshore: Behavior and surface tension measurements. *J. Geophysical Research* **112**(C12001), (2007) (doi:10.1029/2007JC004108).

87 Reynolds, O. An experimental investigation of the circumstances which determine whether the motion of water shall be direct or sinuous, and of the law of resistance in parallel channels. *Philosophical Transactions of the Royal Society of London*, **174**, 935–982 (1883).

88 Graham, A. Aeration due to breaking waves. Part II: Fluxes. *Journal of Physical Oceanography*, **34**(5), 1008–1018 (2004).

89 Wise, D. L. & Houghton, G. The diffusion coefficients of ten slightly soluble gases in water at 10-60 °C. *Chem. Eng. Sci.*, **21**, 999–1010 (1966).

90 Jähne. B., Heinz, G. & Dietrich, W. Measurement of the diffusion coefficients of sparingly soluble gases in water. *J. Geophysical Research-Oceans*, **92**(C10), 10767–10776 (1987).

91 Deane G. B. & Stokes, M. D. Scale dependence of bubble creation mechanisms in breaking waves. *Nature,* **418**, 839-844 (2002).

92 Gemmrich, J.R. & Farmer, D.M. Near-surface turbulence and thermal structure in a wind-driven sea. *Journal of Physical Oceanography*, **29**(3), 480–499 (1999).

93 Thorpe, S.A., Osborn, T.R, Farmer, D. M. & Vagle, S. Bubble clouds and langmuir circulation: observations and models. *Journal of Physical Oceanography*, **33**(9), 2013–2031 (2003).

94 Thorpe, S.A. Langmuir circulation. *Annual Review of Fluid Mechanics*, **36**, 55–79 (2004).

95 Blackford, J., *et al.* Detection and impacts of leakage from sub-seafloor deep geological carbon dioxide storage, *Nature Climate Change* **4**(11), 1011-1016 (2014) (doi: 10.1038/nclimate2381).

96 Berges, B. J. P., Leighton, T. G. & White, P. R., Passive acoustic quantification of gas fluxes during controlled gas release experiments. *International Journal of Greenhouse Gas Control*, **38**, 64-79 (2015) (doi:10.1016/j.ijggc.2015.02.008).

97 Brooks, I.M., *et al.* Physical Exchanges at the Air Sea Interface: UK SOLAS Field Measurements. *Bulletin of the American Meteorological Society*, 90, 629-644 (2009) (doi: 10.1175/2008BAMS2578.1) (with electronic supplement on pages ES9-ES16, doi: 10.1175/2008BAMS2578.2).

98 Upstill-Goddard R. *et al.* UK-SOLAS Cruise Report: RRS Discovery Cruise D320, 16 June - 18 July, 2007: DOGEE-II: air-sea gas exchange in the Atlantic Ocean. *Technical report, Open Research Group, School of Marine Science and Technology, University of Newcastle upon Tyne,* (2007).

99 Yelland, M. J., Pascal, R. W., Taylor, P. K., & Moat, B. I. AutoFlux: an autonomous system for the direct measurement of the air-sea fluxes of

CO_2, heat and momentum. *J. Operational Oceanography*, **2**(1), 15–23 (2009).

100 Smith, S. D. Coefficients for sea-surface wind stress, heat-flux, and wind profiles as a function of wind-speed and temperature. *J. Geophysical Research-Oceans*, **93**(C12), 15467–15472 (1988).

101 Thorpe, S.A. Cure, M.S., Graham, A. & Hall, A. J. Sonar observations of Langmuir circulation and estimation of dispersion of floating particles. *J. Atmospheric and Oceanic Technology*, **11**(5), 1273-1294, (1994).

102 Leibovich, S. The form and dynamics of Langmuir circulations. *Annual Review of Fluid Mechanics*, **15**, 391–427 (1983).

103 Smith, J.A. Observed growth of Langmuir circulation. *J. Geophysical Research-Oceans*, **97**(C4), 5651–5664 (1992).

104 Callaghan, A., de Leeuw, G., Cohen, L. & O'Dowd, C. D. Relationship of oceanic whitecap coverage to wind speed and wind history. *Geophysical Research Letters*, **35**(23), L23609 (5 pages) (2008) (doi: 10.1029/2008GL036165).

105 Cummings, P. D. & Chanson, H. An experimental study of individual air bubble entrainment at a planar plunging jet. *Chemical Engineering Research and Design*, **77**(A2), 159–164 (1999).

106 Ervine, D.A., McKeogh, E. & Elsawy, E.M. Effect of turbulence intensity on the rate of air entrainment by plunging water jets. *Proceedings of the Institution of Civil Engineers Part 2-Research and Theory*, **69**(JUN), 425–445 (1980).

107 Farmer, D. M. & Vagle, S. Waveguide propagation of ambient sound in the ocean-surface bubble layer. *J. Acoust. Soc. Am.* **86**, 1897–1908 (1989).

108 Phelps, A. D. & Leighton, T. G. Oceanic bubble population measurements using a buoy deployed combination frequency technique. *IEEE J. Oceanic Eng.* **23**, 400–410 (1998) (doi:10.1109/48.725234).

FIGURE CAPTIONS

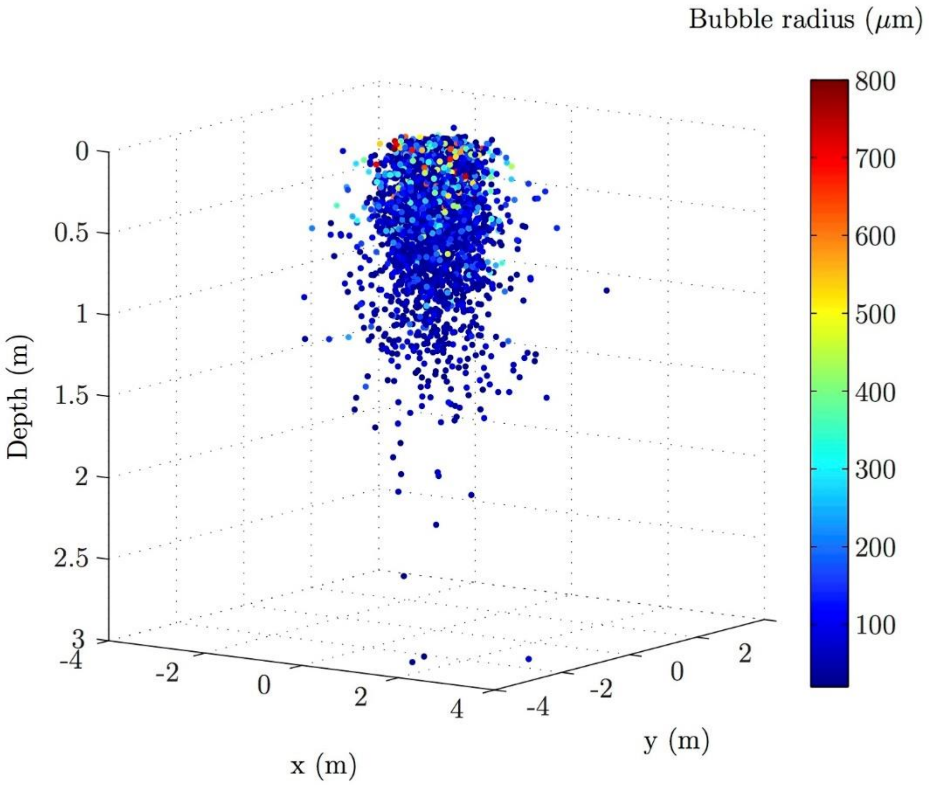

Figure S3.1 A three-dimensional view of the Thorpe model bubble cloud once steady state has been reached. Each second, 10,000 bubbles ranging in radius from 10 µm to 10 mm were input at the surface. The turbulent diffusion coefficient was 0.01 m^2 s^{-1}, and water temperature was 10 $^{\circ}$C.

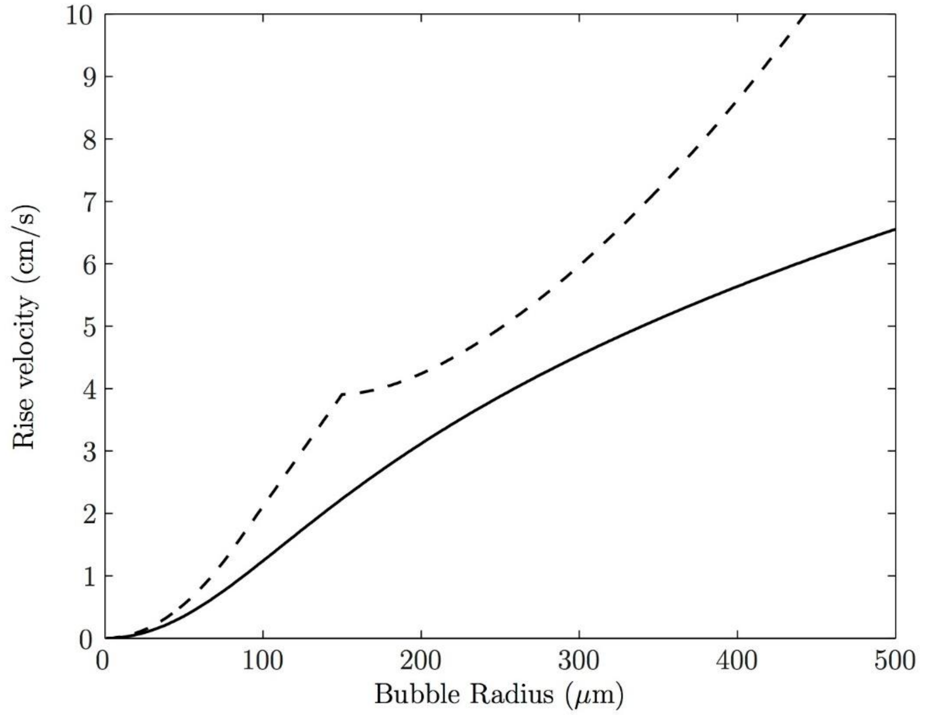

Figure S3.2 Bubble rise speeds for hydrodynamically clean (dashed line) and dirty (solid line) bubbles, according to Thorpe[78] and Levich[79].

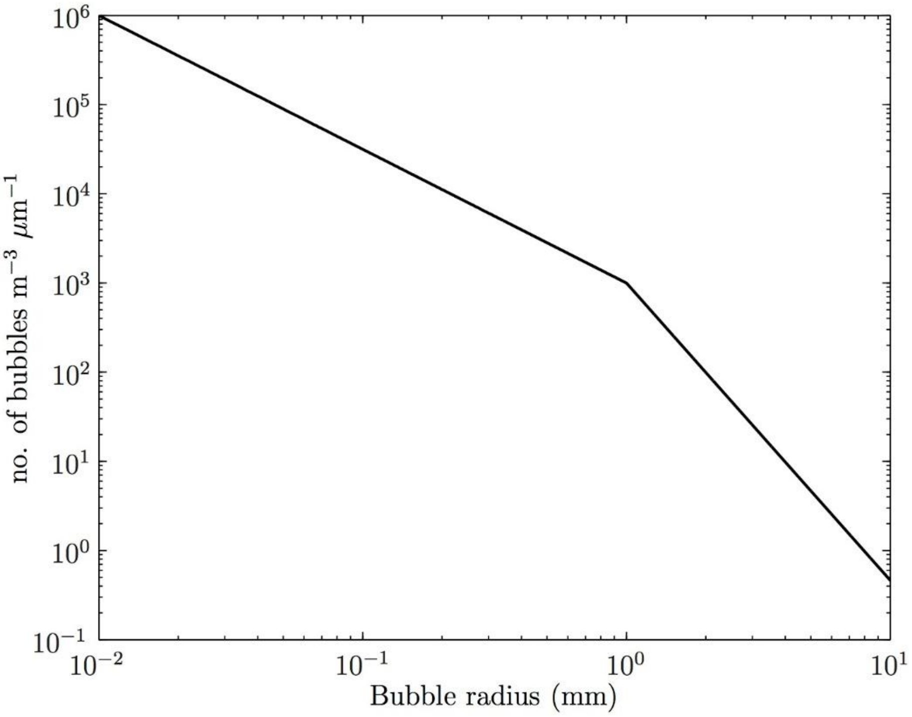

Figure S3.3 Best fit lines extrapolated for data measured under a breaking wave by Deane and Stokes[91] and used here for the initial bubble size distribution. For bubbles smaller than 1 mm, the number varies as $R_0^{-10/3}$. For bubbles larger than 1 mm, the number varies as $R_0^{-3/2}$.

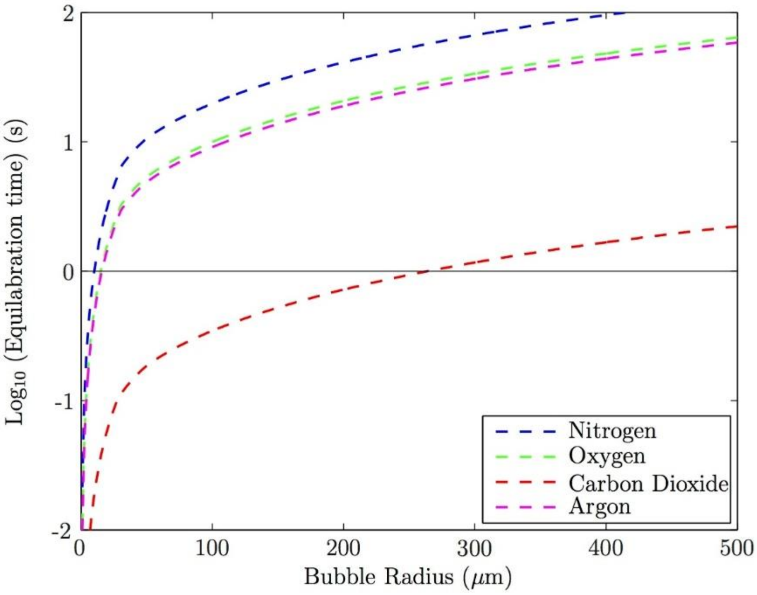

Figure S3.4 The equilibration times for the four gases for a range of bubble radii. The bubbles modelled here are hydrodynamically dirty.

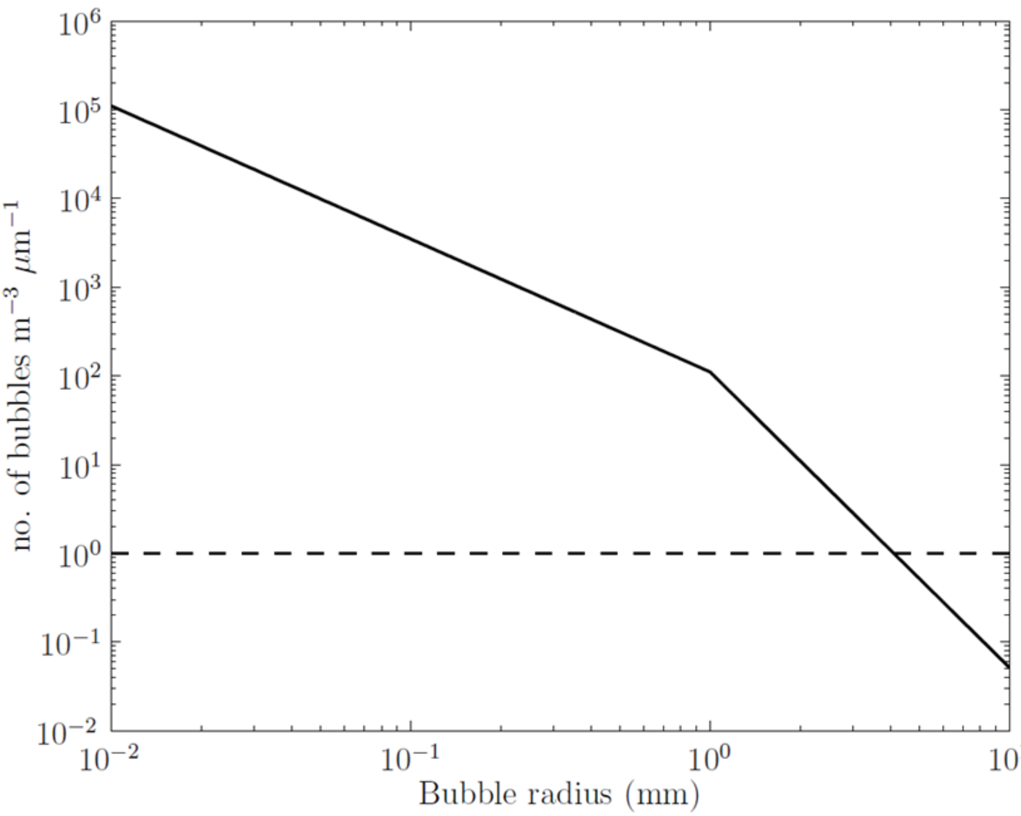

Figure S3.5 The input population taken from Figure S3.3 and reduced by a factor of 100. A horizontal line drawn when the vertical axis equals unity marks the point below which rounding errors become significant.

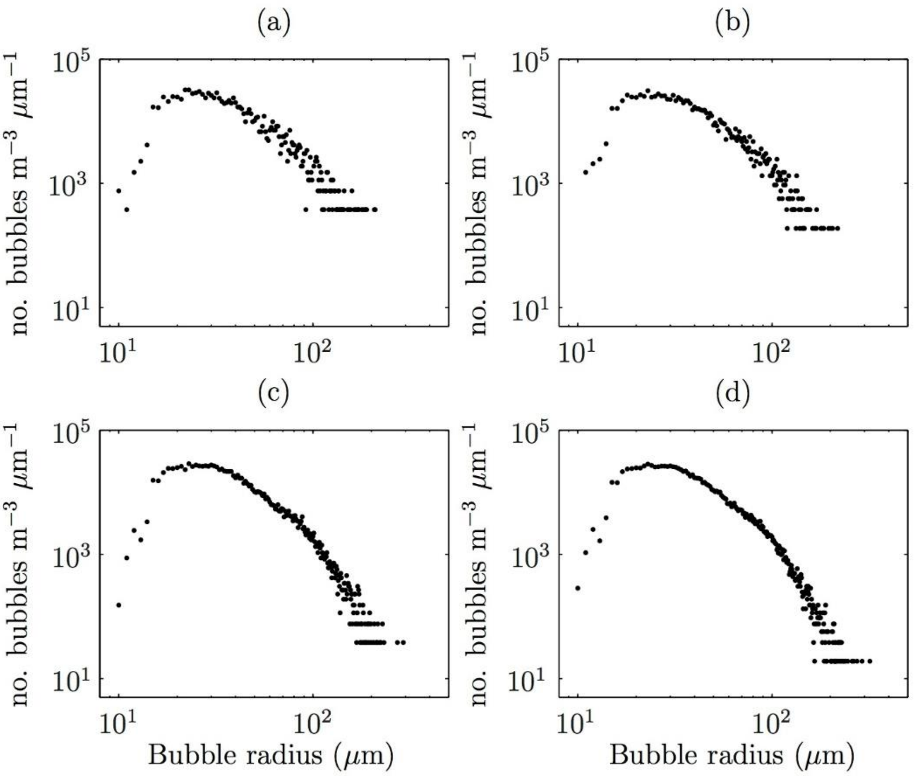

Figure S3.6 Bubble size distributions produced by four identical model runs. The number of bubbles in the input population was the only variable. Panel (a) used 50,000 bubbles for the input, (b) used 100,000, (c) used 500,000 and (d) used 1 million bubbles. Each time, the size distribution is scaled up by the factor with which the input population was initially scaled down.

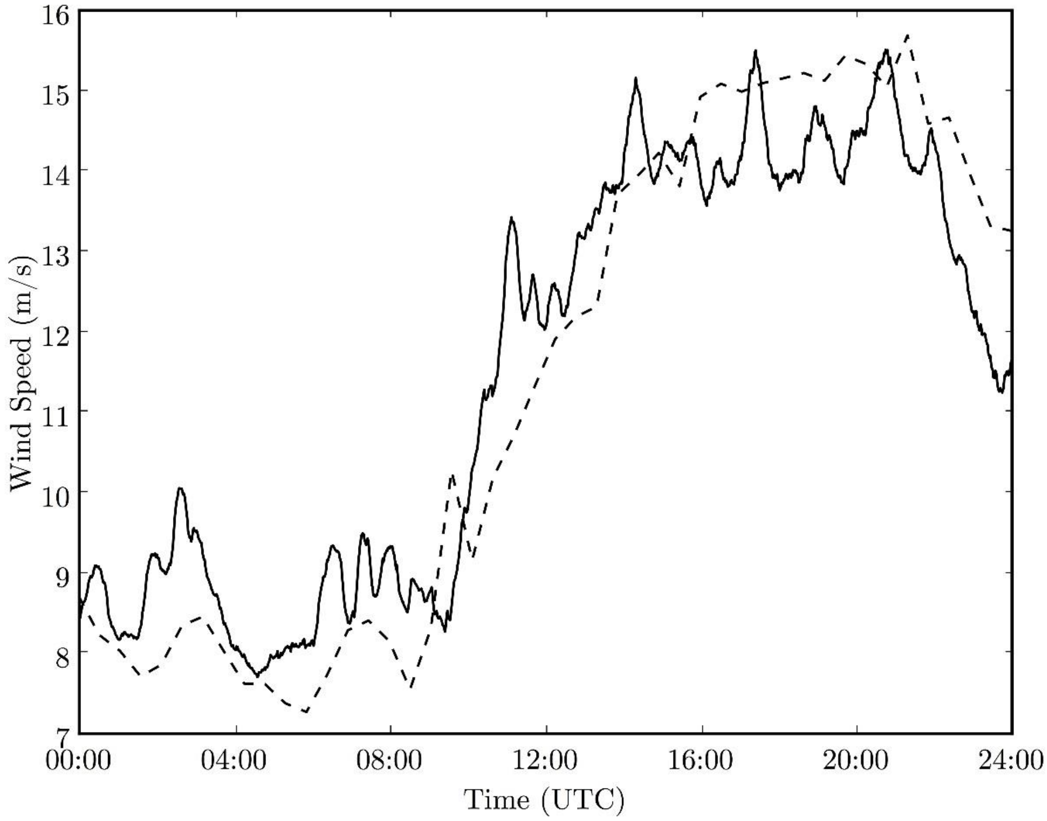

Figure S3.7 Wind speeds as measured by AutoFlux on the ship (the solid line) and the ASIS-2 buoy, located near the spar buoy (dashed line). Both datasets have been corrected to 10 metres using techniques outlined by Smith[100]. The ASIS-2 data was kindly supplied by Professor William Drennan of RSMAS, University of Miami.

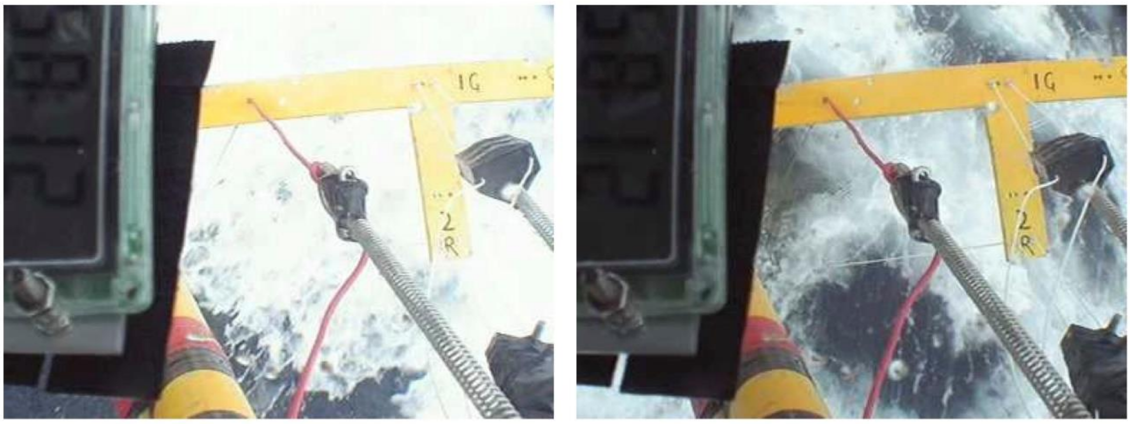

(a) (b)

Figure S3.8 Still images taken from the downward looking video camera in the dome on top of the buoy. (a) Shows the breaking wave hitting the buoy and (b) shows the sub-surface bubble clouds resulting from the breaking wave. The images were taken on 29th June 2007 at 17:58 GMT. Images courtesy of Robin Pascal of the National Oceanography Centre, Southampton.

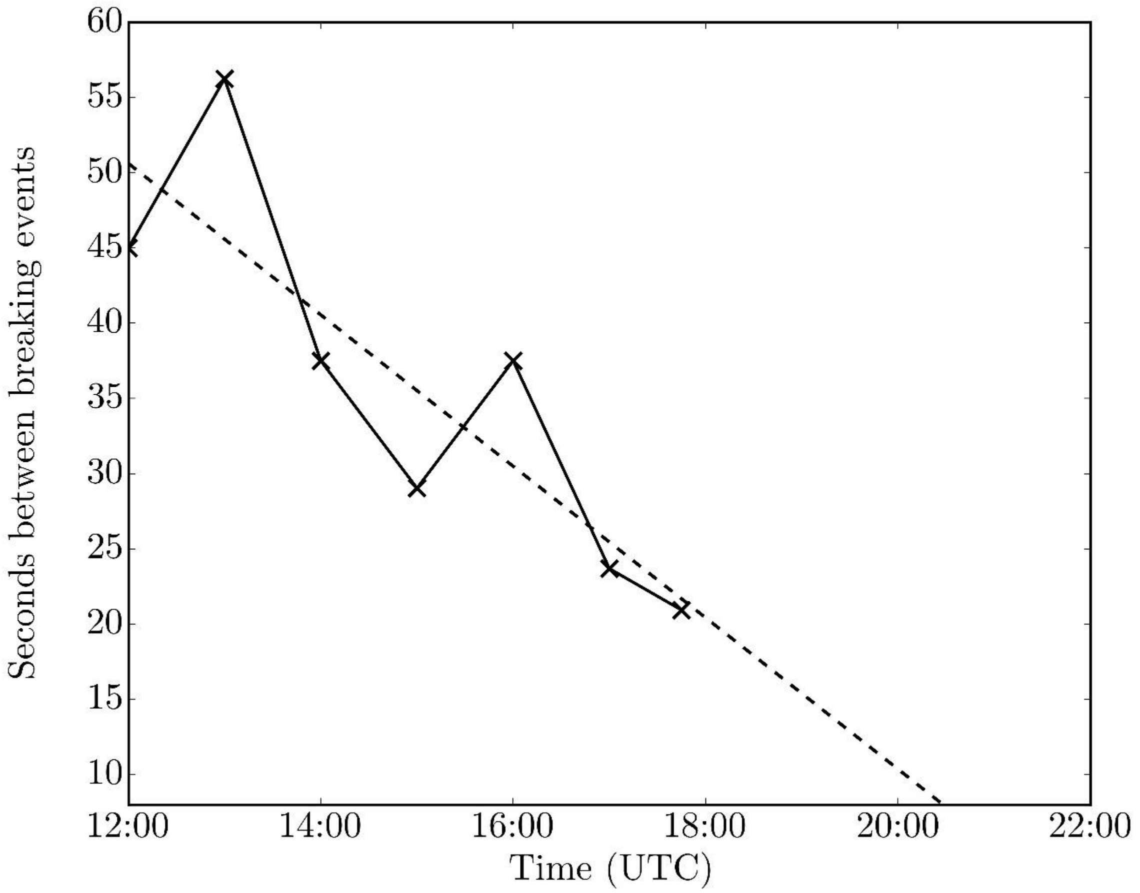

Figure S3.9 The number of seconds between each breaking event (solid black line) between noon and 18:00 on 29th June 2007, the day of measurement on the second deployment of D320. Each point on the graph represents the mean of a 15-minute sample. The dashed line is a linear fit to the video data.

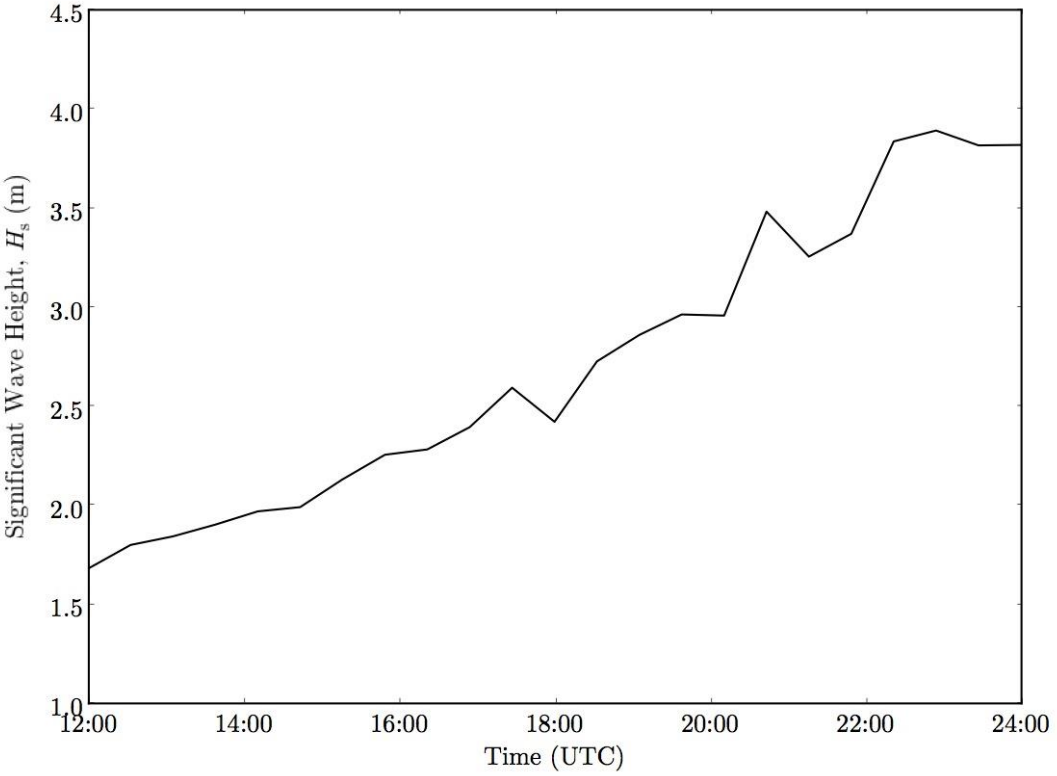

Figure S3.10 The significant wave height measured by the nearby ASIS-2 buoy. ASIS-2 data was kindly supplied by Professor William Drennan of RSMAS, University of Miami.

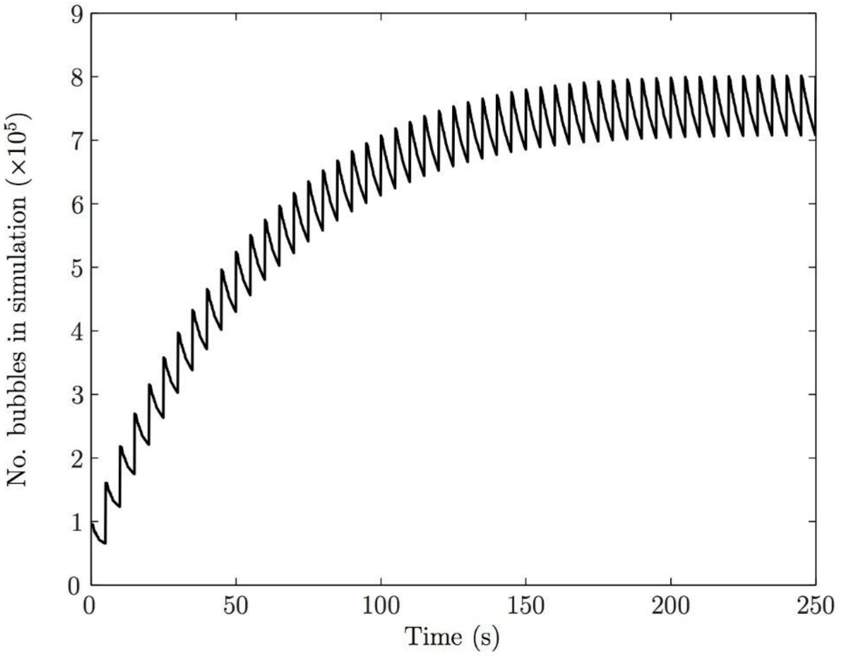

Figure S3.11 A plot of the number of bubbles in the simulation through time. The cloud has clearly reached steady state by the end of the model run. The curve is toothed because of the bubble input every 5 seconds.

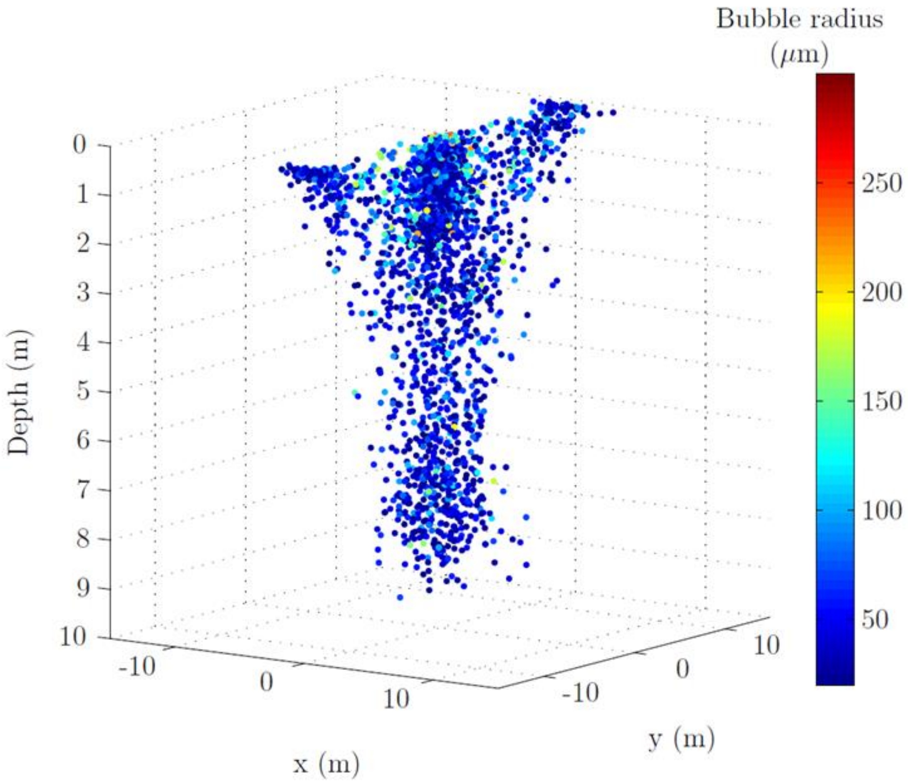

Figure S3.12 The bubble cloud at the end of the model run. The helical flow of the Langmuir cells can be seen. The population shown here is from a run with 100,000 bubbles in the input population, and only 1 in every 100 bubbles is plotted. Input parameters can be seen in Table S3.3.

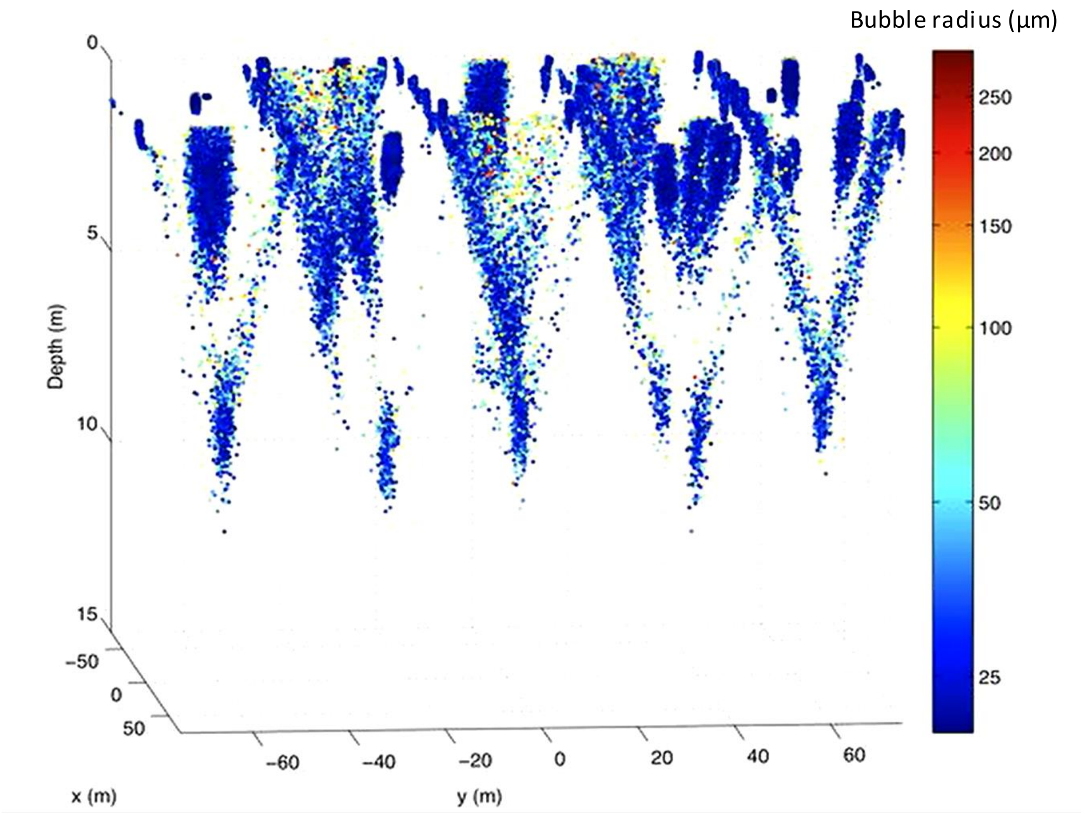

Figure S3.13 The simulation is run to cover multiple breaking waves over a field containing several Langmuir cells. The result shows how the dispersive effect of turbulence on the bubble plumes are countered by the ordering effects of Langmuir circulation and buoyancy. The colour scale indicates bubble radius in microns. This video is a frame from the accompanying supplementary video entitled ‘Langmuir animation’.

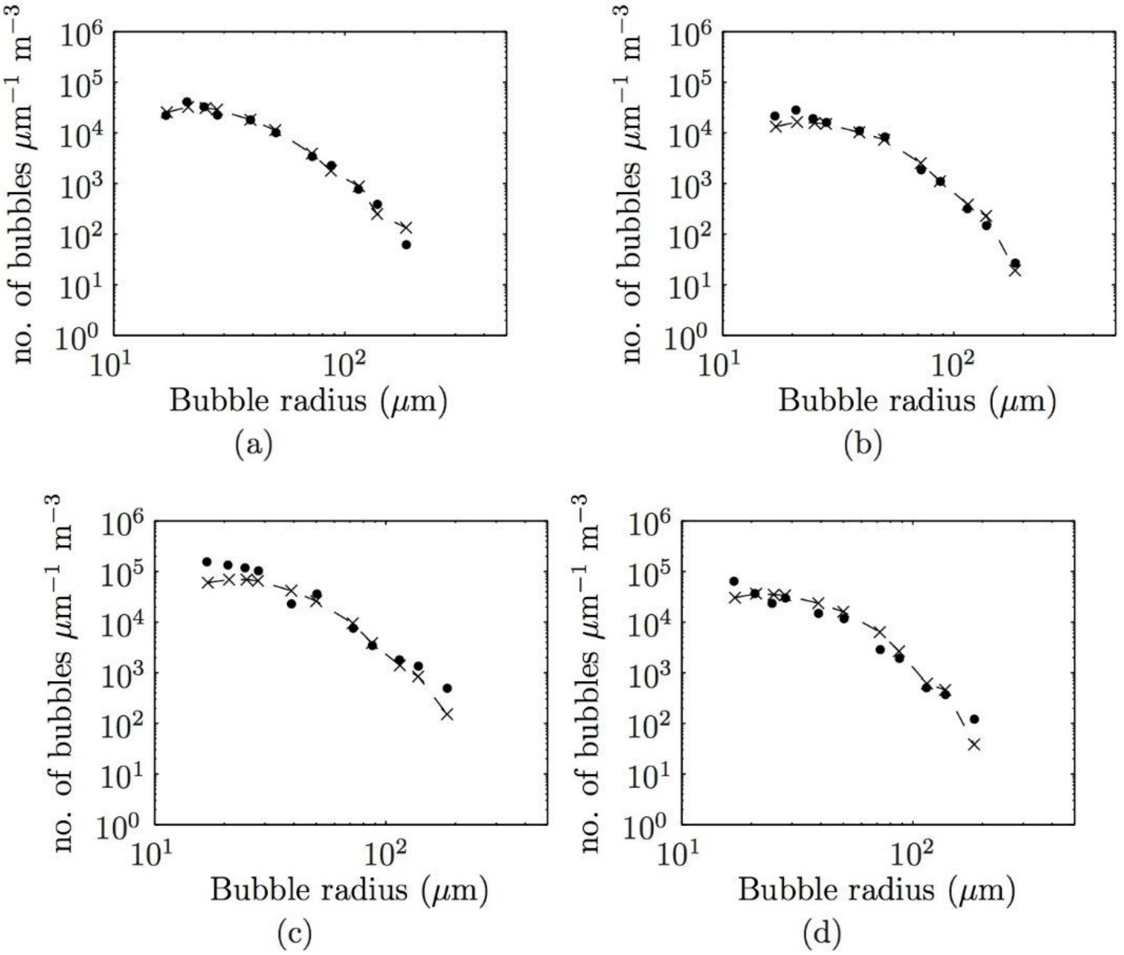

Figure S3.14 Best fits between the minimum and maximum bubble size distributions and the model results with scaled input populations. Panels (a) and (b) show the minimums and 1.15 m and 2 m depths respectively. Panels (c) and (d) show the maximums at 1.15 m and 2 m depths respectively. In each case, the black dots show the measurements and the dashed line and crosses show the model results.

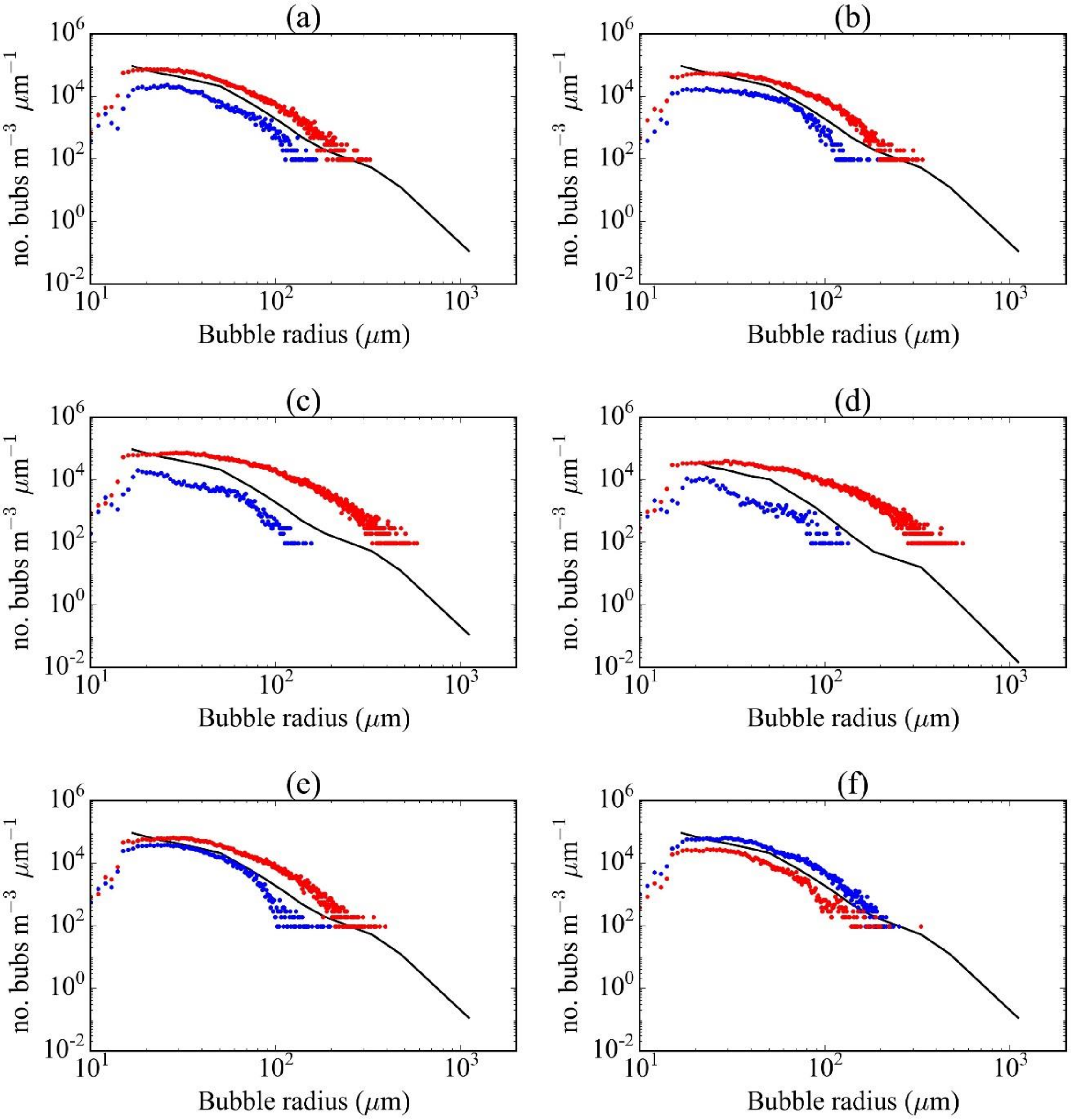

Figure S3.15 The sensitivity of the model output to varying parameters at 1.15 metres depth. In each subfigure, the solid line is the original best fit solution, the red dots represent an increase in each parameter and the blue dots represent a decrease in each parameter. Panel (a) shows the results from varying the maximum Langmuir velocity (0.1 and 0.25 m s^{-1}), panel (b) is the length of the initial jet (0.6 and 1.5 s), panels (c) and (d) are the initial jet velocity at 1.15 and 2 m respectively (0.2 and 1 m s^{-1}), panel (e) is the bubble insertion depth (0 and 0.15 m) and panel (f) is the time between breaking waves (5 and 15 s).

	Nitrogen	Oxygen	Carbon dioxide	Argon
Fraction in dry air	0.7809	0.2095	350×10^{-6}	9.3×10^{-3}
Coefficient of diffusion, D_{mol} ($\times 10^{-9}$ $m^2 s^{-1}$)	1.8	1.7	1.3	1.3
Schmidt Number, Sc	772	818	1069	818
Solubility, S ($\times 10^{-6}$ mol m^{-3} Pa^{-1})	6.29	13.0	445	14.2

Table S3.1: Standard values used for certain parameters in the model. Diffusion coefficient values are taken from Wise and Houghton[89] and Jahne *et al.*[90]

Parameter	Value	Units
Acceleration due to gravity	9.81	$m\ s^{-2}$
Kinematic viscosity	1.39×10^{-6}	$m^2\ s^{-1}$
Molar gas constant	8.314	$J\ mol^{-1}\ K^{-1}$
Bubble surface tension coefficient	3.6×10^{-2}	$N\ m^{-1}$
Water density	1025	$kg\ m^{-3}$
Atmospheric pressure	101325	Pa

Table S3.2: The standard values used for some of the parameters in the model.

Parameter	Value	Units
Turbulent diffusion coefficient	0.01	$m^2\ s^{-1}$
Maximum down welling/upwelling velocity	0.185	$m\ s^{-1}$
Initial jet velocity	0.42	$m\ s^{-1}$
Length of initial jet	1.1	s
Bubble insertion depth	0.035	m
Time between breaking waves	8	s

Table S3.3: The final parameters used in the gas flux model to produce the populations shown in Figure 4 of the main paper and the results summarized in Table 1 of the main paper.
